# Advancing understanding of developmental coordination disorder in children: data from the literature

**DOI:** 10.3389/fnhum.2026.1779776

**Published:** 2026-06-03

**Authors:** Valentina Napoli, Laura Castellini, Andrea De Stefano, Arianna Marzocca, Angelo Dimalta, Domenico Marco Romeo, Claudia Brogna

**Affiliations:** 1 Pediatric Neurology Unit, Università Cattolica del Sacro Cuore, Rome, Italy; 2 Pediatric Neurology Unit, Fondazione Policlinico Universitario “A. Gemelli”, IRCCS, Rome, Italy

**Keywords:** DCD, EEG, eye tracking, gait analysis, neurodevelopmental disorder, neuroimaging

## Abstract

Developmental Coordination Disorder (DCD) should be considered as a multifaceted neurodevelopmental disorder characterized by extensive cerebral structural, functional, and connectivity patterns. DCD is commonly associated with other developmental conditions, including attention deficit/hyperactivity disorder (ADHD), autism spectrum disorder (ASD), learning disabilities (LD), speech-language delays and emotional and behavioral problems. This comprehensive review, using PRISMA method, analyzes in 72 studies the relation between neurophysiological and neurobiological principles of pediatric DCD (from 0 to 18 years), through several functional findings including EEG, functional neuroimaging (fNIRS and fMRI), eye-tracking, and gait analysis. We report data related to DCD associated with other neurodevelopmental comorbidities (as well as ADHD and ASD) in order to verify if there is some differences in the brain areas involved. There were excluded all the case reports, reviews, analyzing adult subjects or non-written in English. The results showed that in children with DCD structural, functional, and connectivity abnormalities in multiple brain areas can be found, compared with healthy individuals, showing atypical activation in the frontal lobes, parietal lobes, cerebellum, and basal ganglia during cognitive and sensorimotor processing. The most brain areas involved were the DLPFC, the right inferior frontal gyrus (IFG), the posterior cerebellum, the supplementary motor area (SMA). Furthermore, these studies highlight that comorbidity with ADHD and ASD are associated with a more severe neurobiological signature, even in a heterogeneous mode. Our findings proposed a holistic presentation of the DCD as a condition in which the cerebral network pattern of functioning might result in a complex functional phenotype. All these consequences can impact on the cognitive and attentional domains, and consecutively on the child’s mental health at risk of internalizing disorders and social disengagement. Therefore, implementing multimodal strategies enabling the integration of neuroimaging, neuropsychological data, and clinical observation, could improve the early detection of biomarkers and the development of *ad hoc* and individualized therapeutic approach, optimizing long-term endpoints.

## 1 Introduction

Developmental Coordination Disorder (DCD) is a neurodevelopmental disorder characterized by persistent difficulties in motor skills that interfere with daily life, with early onset and not attributable to other neurological or sensory conditions (American Psychiatric Association, 2013). The children with DCD are usually described as “clumsy” or “inaccurate” when performing; the gross and/or fine motor skill deficits interfere with activities of daily living or in school productivity/academic achievements (e.g., dressing, tying shoes or going down stairs, playground skills, handwriting, gym activities). The International Classification of Diseases (ICD)-11, adopted by the World Health Organization, also emphasizes the relationship with intellectual functioning, requiring that these motor skills be below the level expected not only for chronological age but also for the individual’s intellectual functioning. It further expands the exclusion criteria considering neurological and sensory disorders, musculoskeletal and connective tissue conditions. Lastly, it specifies that the impact of the disorder must concern not only daily and school activities, but also potential work and recreational activities. From an epidemiological perspective, DCD has an average prevalence of 5% in the general pediatric population (Li et al., 2024). Rates are higher in males (7, 95% CI: 4–10%) compared to females (4, 95% CI: 3–7%), and vary by geographic area: 4% in Asia, 2% in Europe, and 6% in North America (Li et al., 2024).

Among the main risk factors documented for DCD, the literature and international recommendations (Blank et al., 2019) identify preterm birth (< 37 weeks) and very low birth weight (< 1,250 g). The meta-analysis by Li et al. (2024) reports a prevalence of 18% (95% CI: 8–31%) in preterm births, while in children with very low birth weight the prevalence reaches to 31% (95% CI: 14–50%), compared to 6% (95% CI: 4–7%) term born babies. Furthermore, recent studies have suggested a possible role of generalized joint hypermobility as an additional risk factor. In particular, Romeo et al. (2022) reported that, in a cohort of low-risk preterm preschool children, approximately 20% presented joint hypermobility and showed significantly lower Movement Assessment Battery for Children (M-ABC-2) scores compared to peers without hypermobility. This review also highlights that the co-occurrence between DCD and hypermobility, across different studies, varies between approximately 33 and 48% (Romeo et al., 2022). The Peabody Developmental Motor Scales (PDMS-2) (preschool children) and the Developmental Test of Visual-Motor Integration (Beery VMI) can provide useful information on the nature of the movement difficulties. The M-ABC 2 is the most important test uses to assess manual dexterity, aiming and grasping skills, and balance (static and dynamic) in children aged 3 to 16 years.

Despite the significant public health impact of DCD, markedly affecting school performance, social participation, and mental health, it remains underdiagnosed and under-researched from a neurobiological perspective (Blank et al., 2019). Evidence from neuroimaging studies is still limited, characterized by small sample sizes and considerable methodological heterogeneity (Yıldırım et al., 2021; Zwicker et al., 2012). Magnetic resonance imaging (MRI) studies have reported differences in brain regions involved in planning and motor control in children with DCD; however, small sample sizes and methodological variability make it necessary to further investigate these findings before drawing definitive conclusions (Brown-Lum and Zwicker, 2017). Recent neuroimaging studies have provided important insights into the structural and functional alterations in the brains of children with DCD, such as cortical thinning in the right medial orbitofrontal cortex and changes in white matter organization, particularly in sensorimotor tracts, suggesting a compromised neural connectivity across the brain (Wilson et al., 2017). Furthermore, functional neuroimaging studies, including electroencephalography (EEG) and functional magnetic resonance imaging (fMRI), have shown hypoactivation in key brain areas, such as the cerebellum, prefrontal cortex, and parietal lobes, which are crucial for motor control and internal modeling. These findings suggest that children with DCD not only have motor difficulties but also cognitive and neural dysfunctions that complicate motor control (Zwicker et al., 2012; Debrabant et al., 2013; Kashiwagi et al., 2009; Querne et al., 2008).

Considering the frequency, persistence of symptoms, and long-term consequences, international recommendations emphasize the importance of early diagnosis and targeted interventions, not only to improve motor performance but also to promote social participation and prevent secondary psychiatric complications (Blank et al., 2019) as well as obsessive-compulsive disorder, anxiety including social phobia (Pratt and Hill, 2011). Additionally, thinking about the trajectory of development, the emotional distress experienced during the childhood marked by DCD would affect the mood during the adolescence (Wagner et al., 2012) and subsequently affecting QoL (Tal-Saban et al., 2012; Kirby et al., 2014), until the adulthood age (Engel-Yeger and Engel, 2023).

The present study aims to provide an integrated comprehensive review of the available neurophysiological and instrumental evidence on children with DCD and the pattern of the brain networks interested. Specifically, it examines contributions from EEG, functional near-infrared spectroscopy (fNIRS), fMRI, single-photon emission computed tomography (SPECT), eye-tracking, and gait analysis, with the goal of outlining a more unified framework of the neurobiological correlates of the disorder.

Furthermore, although gait abnormalities are among the most frequently reported difficulties in clinical practice, the scientific literature has long remained fragmented, and recently only the first systematic syntheses has been published, that rigorously compare gait in children with and without DCD, revealing differences in both kinematic and spatiotemporal parameters (Smith et al., 2021). This gap justifies the inclusion of gait analysis within the present review and underscores its relevance for a more comprehensive understanding of the neuro-motor profile of DCD.

## 2 Materials and methods

### 2.1 Search criteria

**Figure 1:**
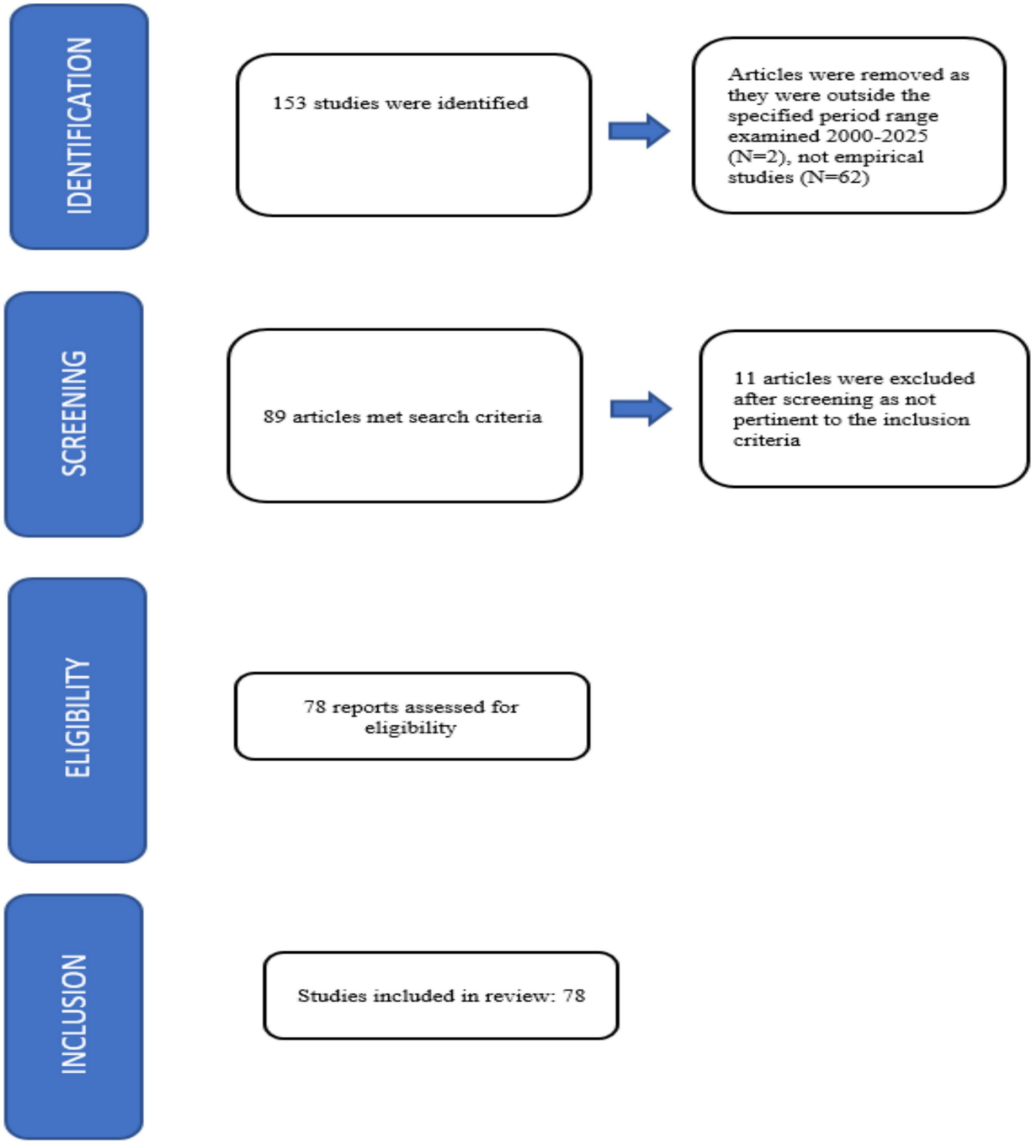
Flow chart for process of according to PRISMA.

The review was carried out according to the guidelines Preferred Reporting Items for Systematic Reviews and Meta-Analyses (PRISMA; see Figure 1).

The research was conducted using the following databases: PUBMED, MEDLINE, Embase, and Scopus.

The search terms used were “DCD,” combined with “children,” “EEG,” “Functional MRI,” “single photon emission computed tomography (SPECT),” “functional Near-Infrared Spectroscopy (fNIRS),” “eye tracking,” “gait analysis” “neurodevelopmental disorder,” “Attention-Deficit/Hyperactivity Disorder (ADHD),” “Autism spectrum disorder (ASD).” Duplicates were excluded prior to reference retrieval. Abstracts for each reference were obtained and screened according to a predefined format listing the inclusion and exclusion criteria.

### 2.2 Inclusion criteria

Studies were included if they were written in English or if a suitable English translation was available, and if they were human-based. All studies were initially selected by identifying the presence of a clinical association between “DCD” and at least one of the following terms:,” EEG,” “functional MRI,” “SPECT,” “eye tracking,” “gait analysis” “neurodevelopmental disorder,” “Attention-Deficit/Hyperactivity Disorder (ADHD),” “Autism spectrum disorder (ASD).” Only articles published between 2000 and 2025 were included. The age range considered was from 0 to 18 years.

### 2.3 Exclusion criteria

The exclusion criteria for this review were clearly defined before the screening process began. Specifically, during the identification of relevant studies, articles were excluded if they were case reports, reviews, analyzing adult subjects or non-written in English.

### 2.4 Data extraction and analysis

The title and abstracts of the studies were independently examined for suitability by two authors (V. N., L. C) and critically checked by a third independent reviewer (C. B.); conflicting viewpoints were discussed until a consensus was reached. A total of 153 studies were initially identified; after a review of the full text, 75 were excluded, as they included adult-samples or they were reviewed or as non-English published articles to ensure consistency in data extraction, evaluation and interpretation. The remaining 78 articles were included in the present review (details are reported in in the Supplementary Tables 1, 2).

*Scope and thematic focus*:

The selected studies encompass a variety of neurophysiological and neuroimaging cerebral patterns identified in pediatric DCD, with a specific focus on the following domains that necessitated the inclusion of diverse methodologies (e.g., randomized controlled trials, observational designs, case series) (Supplementary Tables 1, 2):

- Functional Neuroimaging (Fnirs, fMRI and SPECT);
- EEG and High density EEG (Hd-EEG);
- Eye tracking and oculomotor behavior;
- Gait analysis.

## 3 Results

The studies were subdivided in 2 main groups (1) children presenting isolated DCD and (2) children presenting DCD associated with neurodevelopmental disorders as well as ASD and ADHD.

**Figure 2:**
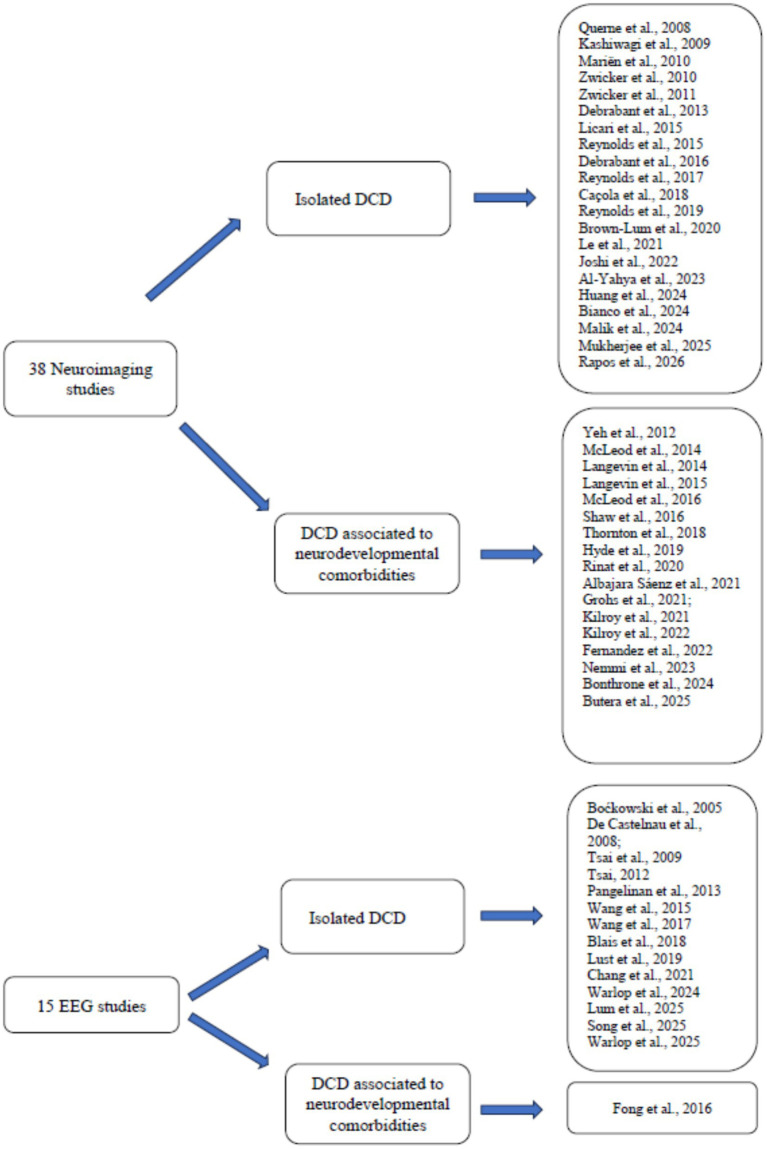
Flow chart “neuroimaging and EEG” studies.

**Figure 3:**
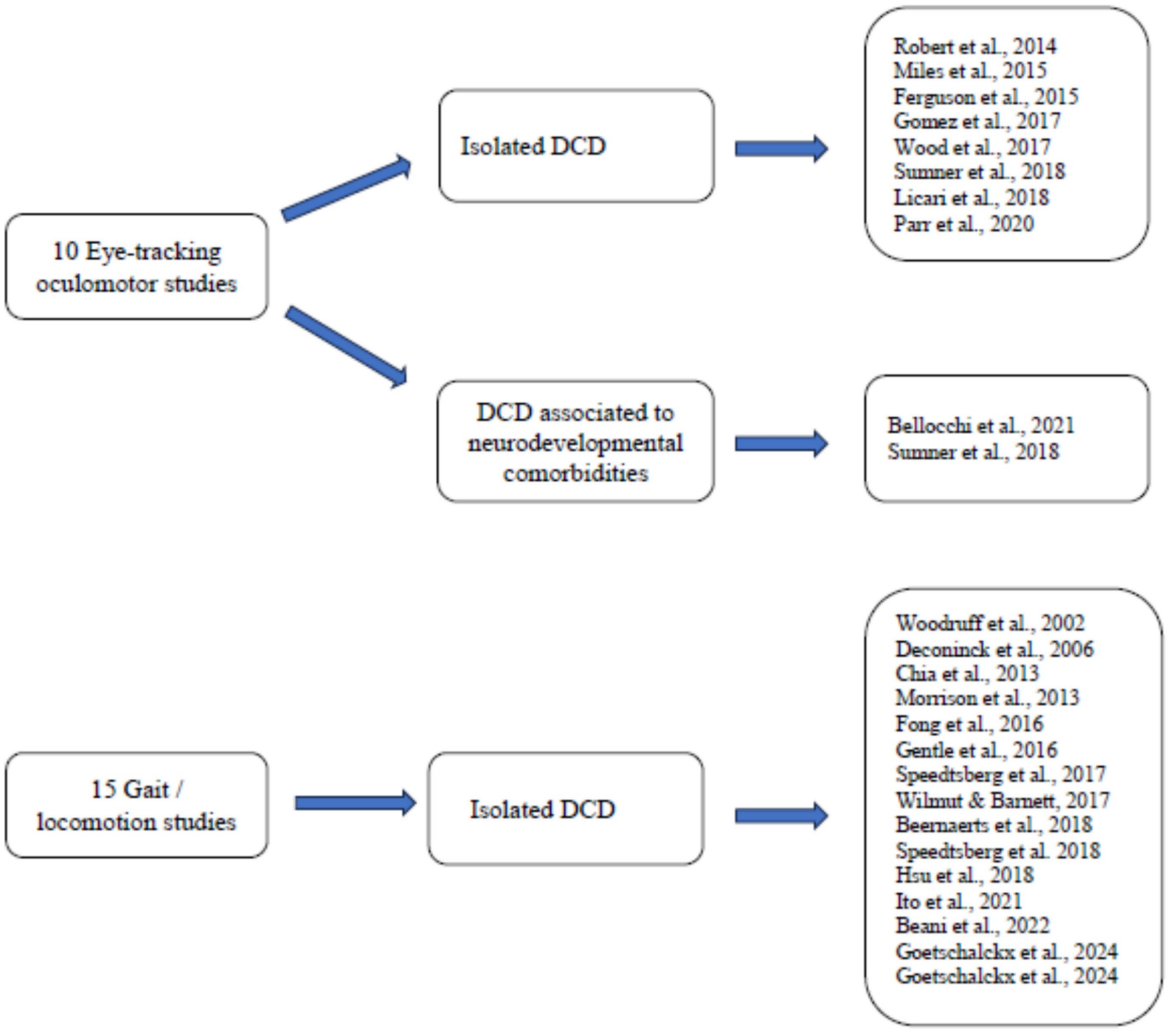
Flow chart “eye-tracking and gait-locomotion” studies.

In Figures 2, 3 has been reported the Flow chart regarding Neuroimaging, EEG, Eye-tracking and Gait-locomotion studies.

### 3.1 Children with isolated DCD

#### 3.1.1 Functional neuroimaging (fNIRS, fMRI, and SPECT)

In recent years, several functional neuroimaging techniques, in particular functional Near-Infrared Spectroscopy (fNIRS) and cerebral functional magnetic resonance imaging (fMRI), have been used to explore the neural correlates of DCD. Early fMRI investigations provided initial evidence that motor difficulties in children with DCD are associated with atypical patterns of brain activation during fine motor practice, even in the absence of marked behavioral differences.

Children with DCD activate different cortical areas using alternative neural networks compared to TDC. Specifically, several studies reported reduced activation of the right DLPFC, the right inferior frontal gyrus (IFG), the posterior cerebellum (Debrabant et al., 2013; Zwicker et al., 2010; Zwicker et al., 2011), the supplementary motor area (SMA) (Joshi et al., 2022), and greater activation in frontal, temporal and parietal regions (Zwicker et al., 2010). Other studies highlighted greater involvement of prefrontal areas, including the DLPFC and Broca’s area, and reduced activation of premotor and cerebellar regions (Caçola et al., 2018; Kashiwagi et al., 2009). Moreover, reduced activation of the attentional network has been observed during inhibition tasks, specifically at the level of the Medial Frontal Cortex (MFC), Inferior Parietal Cortex (IPC) and Anterior Cingulate Cortex (ACC), with greater reliance on the left hemisphere (Querne et al., 2008).

Using fMRI during fine motor skill practice and retention, Zwicker et al. (2010, 2011) demonstrated that children with DCD, despite comparable task performance, exhibited reduced activation of cerebellar–parietal and cerebellar–prefrontal networks compared to typically developing children (TDC); similarly Joshi et al. (2022) (21- > 20) confirmed in DCD reduced activation of the prefrontal cortex, especially in the right middle frontal gyrus, whereas TDC showed an increase activation. A further recent study reported a reduced activation of the left superior parietal cortex during motor control tasks in DCD compared to controls, who instead showed significant activation of the bilateral parietal cortex, the cerebellum and the premotor cortex (Kashiwagi et al., 2009; Huang et al., 2024). Associations between white matter integrity (assessed through Fractional Anisotropy (FA) and Radial Diffusivity (RD) via DTI) and visuomotor performance were investigated by Debrabant et al. (2016). In children with DCD were observed a reduced white matter integrity, reflected in decreased FA and increased RD in the left retrolenticular limb of the internal capsule. Lower FA was associated with poorer visuomotor performance in both DCD and controls in the paper published by Brown-Lum et al. (2020).

These data were also confirmed by structural MRI findings as reported in a T1-weighted structural MRI study including 65 children (24 with DCD and 41 TDC) showing that children with DCD exhibited less efficient implicit motor imagery, assessed using a hand laterality judgment task, and that smaller gray matter volumes in frontal, parietal, and cerebellar regions were associated with poorer motor imagery performance across groups (Mukherjee et al., 2025).

On the other hand, other studies demonstrated a greater activation of different brain areas like as a greater and more widespread recruitment of frontal, parietal and temporal regions, indicating altered neural engagement during motor skill acquisition and consolidation (Zwicker et al., 2010; Zwicker et al., 2011). Al-Yahya et al. (2023) reported greater activation in the left dorsolateral prefrontal cortex (DLPFC) during stepping and dual-task conditions, but less efficient recruitment of the right DLPFC in individuals with DCD by using fNIRS.; A recent task-based fMRI study using task-based BOLD (blood-oxygen-level-dependent) signal changes, including 22 children with DCD and 22 TDC demonstrated that, during a virtual collision-avoidance task, children with DCD showed significantly greater activation in the bilateral visual cortices and the right middle frontal gyrus, whereas no between-group differences were observed during a simpler person-direction task (Rapos et al., 2026). Significantly, response times did not differ between groups, suggesting increased neural recruitment in DCD in the absence of overt motor slowing, consistent with higher cognitive–attentional demands during complex visuomotor decision-making (Rapos et al., 2026). Structural MRI findings have also highlighted gray matter morphological differences in children with DCD. Specifically, a cross-sectional T1-weighted structural MRI study using voxel-based morphometry including 30 children with DCD and 12 TDC reported greater grey matter volume in the left superior frontal gyrus in children with DCD; furthermore greater grey matter volume in frontal and parietal regions was associated with poorer motor performance and higher attentional difficulties (Malik et al., 2024). In addition, fMRI measures showed significant activation of the sensorimotor cortex and the cerebellum during stepping, without main group or time effects (Al-Yahya et al., 2023).

Regarding brain connectivity, children with DCD showed less integrated and less efficient networks and a global efficiency directly correlated with visuomotor performance (Debrabant et al., 2016). At the cerebellar level, reduced nodal efficiency was observed in lobules IV–VI, particularly in the left lobule VI, which was significantly associated with visuomotor performance (Debrabant et al., 2016).

In relation to the mirror neuron system (MNS), fMRI data showed that both DCD and TDC groups activated MNS regions during movement observation. However, in the imitation condition, children with DCD exhibited, in addition to MNS activation, areas of reduced activation in non-MNS regions (thalamus, caudate, posterior cingulate) involved in attentional and motor planning processes (Reynolds et al., 2015). The same author confirmed in a subsequent study (Reynolds et al., 2019) a reduced activation also in the precentral gyrus, the pars opercularis of the IFG and the precuneus in children with DCD. Finally, consistent with these findings, voxel-based morphometry studies have reported reduced relative grey matter volume in motor and attention-related regions in children with DCD, particularly involving frontal and premotor areas, suggesting that structural differences are closely related to motor proficiency and attentional control rather than representing disorder-specific neuroanatomical markers (Reynolds et al., 2017).

In addition, Licari et al. (2015) demonstrated that in DCD, during motor overflow tasks, reduced activation of the left frontal regions (superior and inferior frontal gyri) and greater compensatory involvement of the right postcentral gyrus were observed. Mariën et al. (2010) in a SPECT study demonstrated that children with DCD exhibit significant alterations in cerebellar perfusion and related cortical areas, such as the prefrontal cortex, reinforcing the idea that the cerebellum plays a key role in the pathophysiology of DCD, specifically influencing motor execution and attentional control mechanisms (Mariën et al., 2010). At the cerebellar level, a combined behavioral and structural MRI study investigating procedural learning showed that, after controlling for inattention symptoms, children with DCD did not exhibit the typical implicit learning profile on a serial reaction time task. Procedural learning performance was positively correlated with cerebellar cortical volume, and children with DCD showed a trend toward reduced cerebellar volume compared to TDC (Bianco et al., 2024). A structural MRI study focusing on temporal procedural learning showed that children with DCD exhibited specific impairments in the learning and retention of non-regular auditory temporal sequences, despite the absence of categorical group differences in cortical thickness. Importantly, performance during auditory sequence learning was correlated with cortical thickness in sensorimotor, frontal and parietal regions, suggesting that temporal learning difficulties in DCD are related to functional engagement of fronto-parietal and cerebellar networks rather than to gross structural abnormalities alone (Lê et al., 2021).

#### 3.1.2 EEG

Numerous studies have employed EEG to investigate DCD, identifying differences compared to TDC. Specifically, a recent study (31 children with DCD and 52 TDC) demonstrated an atypical oscillatory profile at resting state, characterized by a delta/alpha imbalance: children with DCD showed increased delta power and reduced alpha power compared to TDC in the eyes-open condition, with the opposite pattern emerging in the eyes-closed condition, suggesting that atypical alpha activity in DCD could be a fundamental brain state, unaffected by the presence or absence of sensory stimulation. No significant differences in beta power were found between the groups. In DCD higher delta power was associated with poorer motor performance, whereas higher alpha power correlated with better motor skills, suggesting that intrinsic brain activity in children with DCD may be related to the severity of motor impairment in affected children (Lum et al., 2025). Another study by Blais et al. (2018) investigated whether motor learning deficits in adolescents with DCD could be explained by reduced integration between cerebral hemispheres. In this study, which included 10 adolescents with DCD and 10 TDC, intra-hemispheric plasticity remained intact, allowing partial improvement after practice, while reduced interhemispheric communication hindered the stabilization of newly acquired skills and fostered motor errors. In addition, De Castelnau et al. (2008) examined the relationship between rhythmic perception, motor execution, and EEG activity in children with DCD. This study demonstrated that children with DCD had higher thresholds in rhythmic discrimination tasks and greater variability in unpaced tapping compared to controls. The presence of auditory rhythmic cues significantly improved accuracy across all groups, including those with DCD, and was associated with increased beta power in fronto-central regions and reduced ipsilateral connectivity. Furthermore, in children with musical training, greater interhemispheric connectivity emerged during auditory-motor synchronization. In another study EEG was recorded from 6- to 12-year-old children with and without DCD (*n =* 14 and 20, respectively) during the performance of a visuomotor drawing task. Although some children with DCD performed outside the typical developmental (TD) landscape, the group developmental trajectory of children with DCD was similar to that of TD children. Despite overall similarities in performance, the engagement of cortical resources differed markedly between children with DCD and their TD counterparts (Pangelinan et al., 2013). Studies on a synchronization/syncopation task, with EEG recording (coherence analysis and evoked potentials), showed that DCD children exhibited major interindividual variability and did not improve performance with repetition. In younger children with DCD, increased frontocentral coherence, enhanced motor preparation components, and prolonged N100 latency were observed in evoked potentials (Boćkowski et al., 2005; De Castelnau et al., 2008). Tsai et al. (2009) further investigated the mechanisms of brain activity in children with DCD using a visuospatial attention-shifting paradigm combined with event-related potentials (ERP). This study revealed that children with DCD showed longer reaction times, deficits in inhibitory response capacity, and distinct electrophysiological characteristics, including longer cue-P3 and target-N1 latency, smaller target-P3 amplitude, and a prolonged interval between N2 and the motor response (N2-RT). These findings suggest that DCD children exhibit deficits in visual processing, inhibitory control, and cognitive-to-motor transfer. Furthermore, later Tsai et al. (2012) examined the effects of chronic aerobic exercise on children with DCD during a visuospatial working memory (VSWM) task, finding significant improvements in accuracy rates and neuroelectric indices, including enhanced P3 amplitudes during both encoding and retrieval phases. Using time–frequency EEG analysis during a visuospatial working memory task, school-aged children with DCD (*n =* 29) showed poorer behavioral performance compared with typically developing peers (*n =* 29), together with reduced modulation of theta and alpha oscillations during encoding and maintenance phases. In particular, diminished pre-stimulus alpha activity was associated with poorer task performance, indicating impaired neural mechanisms supporting the maintenance of task-relevant information in DCD (Wang et al., 2017). The study by Wang et al. (2015) measured EEG oscillations associated with attentional orienting, revealing altered frontal midline theta activity in children with DCD compared to typically developing peers (Wang et al., 2015).

More recently children with DCD showed markedly attenuated modulation of beta power over motor areas during sequence learning, while theta/alpha modulations over occipital regions remained preserved (Lum et al., 2025). In addition, a study reported reduced functional connectivity across multiple frequency bands at rest and during a visuomotor task in boys with DCD, along with atypical right-lateralized beta connectivity in motor regions, suggesting altered network organization underlying motor difficulties (Song et al., 2025).

Furthermore, children with DCD showed significantly less “mu” rhythm desynchronization than typically developing peers during both action observation and execution (Licari et al., 2015). This pattern of activity correlated with poorer attention and motor skills, indicating diminished perceptual–motor specificity. Similarly, EEG evidence from another study showed diminished “mu” rhythm modulation during both action observation and execution in children with DCD, supporting atypical activation of the mirror neuron system in this population (Lust et al., 2019). In addition, an EEG study in young children at risk for DCD showed poorer duration and rhythm perception together with delayed P3a latency in response to temporal deviants, indicating less efficient neural processing of auditory timing (Chang et al., 2021).

Beyond oscillatory activity, EEG can also provide event-related markers relevant to motor learning mechanisms in DCD. A recent study Warlop et al. (2025) examining visuomotor adaptation employed the error-related negativity (ERN), an event-related potential indexing sensory prediction error processing. Children with DCD showed reduced ERN amplitudes and greater directional errors during early adaptation, suggesting diminished sensitivity to prediction errors. Although performance stabilized with extended practice, they did not reach the accuracy of typically developing peers, indicating that motor control limitations and a noisier sensorimotor system—rather than a primary learning deficit—may constrain performance. This study highlights the contribution of non-oscillatory EEG indices to understanding internal model updating in DCD.

Furthermore, EEG evidence indicates alterations in temporal and predictive mechanisms involved in motor tasks in children with DCD: during a visuomotor adaptation task, children with DCD showed reduced sensitivity to sensory prediction errors, indexed by attenuated error-related negativity (ERN) during early learning phases, together with persistently greater directional errors, suggesting that motor control limitations and increased sensorimotor noise may constrain adaptive performance (Warlop et al., 2025). In addition, using an EEG frequency tagging paradigm to assess biological motion perception, children with DCD exhibited reduced neural differentiation between biological and non-biological motion, indicating atypical perceptual processing of biological movement (Warlop et al., 2024).

#### 3.1.3 Eye tracking and oculomotor behavior

Children with DCD show reduced accuracy and slower response times during numerical cognition and visuospatial tasks compared to typically developing children, while still preserving their understanding of numerical linearity (Gomez et al., 2017). Deficits in saccadic inhibition and in maintaining fixation were reported by Sumner et al. (2018a), who observed—using the Eyelink 1,000 system—that children with DCD had greater difficulty sustaining visual attention on a target, showing reduced fixation and pursuit accuracy and a higher number of antisaccade errors compared to their typically developing peers. Conversely, pursuit gain and response preparation in pro- and antisaccade tasks were similar between groups, consistent with previous evidence of impaired smooth pursuit gain (Robert et al., 2014) and visuomanual tracking difficulties under reduced visual feedback (Ferguson et al., 2015). Children with DCD showed impaired inhibitory control and greater oculomotor variability compared to typically developing peers, pointing toward broader deficits in top–down attentional regulation (Sumner et al., 2018a).

In line with these findings, Licari et al. (2018) showed that ocular and inhibitory control difficulties extend to complex visuomotor tasks such as ball catching. Children with DCD displayed a greater number of shorter fixations before ball release and initiated smooth pursuit later during the ball’s flight, although once initiated, pursuit was comparable to controls.

Further supporting the role of gaze strategies in visuomotor performance, Miles et al. (2015) demonstrated that Quiet Eye Training (QET) significantly enhances catching performance in children with DCD. In this study (*n =* 30, age 8–10 years), children receiving QET showed longer quiet-eye durations and marked improvements in catching technique compared to those receiving traditional motor training, with gains persisting at 6-week follow-up. These results converge with later findings by Wood et al. (2017), showing that gaze-focused interventions can improve oculomotor coordination and catching performance, with additional benefits in confidence and psychosocial functioning.

Finally, complementary evidence from real-world locomotor tasks indicates that visuomotor alterations in DCD generalize beyond isolated oculomotor paradigms. In a stair-negotiation study, children with DCD (*n =* 18) exhibited slower and more variable gait, greater reliance on handrails, and significantly increased step-edge clearance variability compared to controls (*n =* 16). Eye-tracking revealed atypical gaze allocation, with greater fixation on handrails and—during descent—a tendency to look further ahead on the staircase, an anxiety-related bias likely reflecting impaired predictive control (Parr et al., 2020).

#### 3.1.4 Gait analysis

So far, only a few gait analysis studies have shown that children with DCD present atypical gait patterns, characterized by high interindividual variability and the absence of a systematic model compared to controls (Woodruff et al., 2002; Chia et al., 2013; Deconinck et al., 2006; Morrison et al., 2013). Specifically, a reduction in peak knee extension and significantly lower extensor moments during the stance phase have been reported, along with reduced ankle power generation and a less efficient running economy, with shorter strides and prolonged stance times (Chia et al., 2013). In another study, Deconinck et al. (2006) further related gait analysis to visual variables, showing that children with DCD rely more heavily on visual information than healthy controls: in fact, under dark conditions, significant reductions in stride length and cadence were observed, along with an increase in medio-lateral oscillation of the center of mass.

In the study of Morrison et al. (2013), an additional variable was analyzed, namely lower limb hypermobility and foot posture, which did not show changes in spatiotemporal variables (cadence, stride length, double support duration), but may nonetheless be relevant for the management of orthoses in these patients. More recently, gait deviation was quantified using the Gait Deviation Index (GDI), showing that school-aged children with traits of DCD (*n =* 23) exhibited significantly greater bilateral asymmetry and lower GDI scores compared with typically developing children (*n =* 23), indicating a more deviant and asymmetric gait pattern during walking (Ito et al., 2021). More recently, children with DCD showed significantly greater spatiotemporal variability and altered interlimb coordination during both walking and running compared with typically developing children, with differences in coordination patterns between lower limbs across gait cycles (Goetschalckx et al., 2024a). In a subsequent experimental walking paradigm involving auditory pacing, children with DCD showed reduced auditory–motor synchronization and altered interlimb coordination when walking to metronomes with different tempi and rhythmic structures, compared with typically developing peers (Goetschalckx et al., 2024b).

Recently, gait studies have shown that children with DCD exhibit reduced local dynamic stability during walking, particularly in the anterior–posterior direction (Speedtsberg et al., 2018). These differences were observed during treadmill walking in a cohort of children with DCD compared with age-matched typically developing controls, indicating greater stride-to-stride variability and lower resistance to small perturbations during steady-state gait (Speedtsberg et al., 2018). In a static postural task, children with DCD (*n =* 9) exhibited significantly greater center-of-pressure (CoP) displacement compared with typically developing children (*n =* 10), with larger rambling and trembling components in both the anterior–posterior and medio–lateral directions across sensory conditions (Speedtsberg et al., 2017). In a task assessing limits of stability, children with DCD showed a significantly reduced maximum excursion in the backward direction compared with typically developing children, which was significantly associated with a higher number of falls in daily life, while no group differences emerged in other directions (Fong et al., 2016b). Consistently, stabilometric assessments showed that children with DCD (*n =* 20) presented greater distance and sway area compared with typically developing children (*n =* 30), although post-hoc comparisons revealed smaller differences between DCD and TD than between DCD and children with cerebral palsy (Beani et al., 2022).

When walking on an irregular terrain, children with DCD (*n =* 35) exhibited significantly greater gait adaptations compared with typically developing controls (*n =* 35). Specifically, the DCD group showed a larger reduction in walking velocity and proportional step length, a greater increase in step with ratio, and a more pronounced forward inclination of the head during locomotion (Gentle et al., 2016).

In a clinical gait coordination context, children with DCD (*n =* 20) showed significantly lower total scores compared with typically developing peers (*n =* 20). Moreover, during pediatric-modified Functional Gait Assessment tasks, children with DCD performed significantly fewer steps before loss of balance across all walking conditions, including narrow-base walking, walking with eyes closed, and backward walking (Hsu et al., 2018).

When negotiating dynamically changing environments involving unexpectedly appearing obstacles, individuals with DCD (*n =* 44) differed significantly from typically developing peers (*n =* 44) in several gait parameters. Participants with DCD initiated path deviations earlier, deviated to a greater extent, and showed higher medio-lateral trunk velocity and acceleration during obstacle circumvention. No group differences were observed in the proportion of trials in which a deviation occurred (Wilmut et al., 2017).

Alongside traditional quantitative approaches, recent studies have also applied qualitative kinematic methodologies to gait analysis. In this context, the use of Qualitative Trajectory Calculus (QTC) allowed the discrimination between children with DCD and typically developing controls by analyzing the relative motion trajectories of lower-limb segments, revealing systematic differences in gait movement patterns between groups (Beernaerts et al., 2019).

### 3.2 Children with DCD associated to neurodevelopmental comorbidities

#### 3.2.1 Functional neuroimaging (fNIRS, fMRI and SPECT)

From a structural perspective, neuroimaging studies investigating cortical and subcortical morphology in children with DCD, ADHD, ASD and their comorbid presentations have yielded heterogeneous and often subtle findings. Across transdiagnostic samples, better motor performance has been variably associated with greater gray matter volume or cortical thickness in frontal and motor regions, although the direction and localization of these associations differ across diagnostic groups. Albajara Sáenz et al. (2021) reported that poorer motor coordination was associated with increased gray matter volume in the right superior frontal gyrus in children with ADHD, and in the right medial frontal gyrus in children with ASD.

Similarly, Langevin et al. (2015) showed that children with comorbid DCD and ADHD exhibit more pronounced and widespread cortical thinning compared with children with isolated DCD or ADHD, particularly involving frontal, parietal, temporal and insular regions, suggesting additive neuroanatomical effects in comorbid conditions. Specifically, cortical thinning in the DCD + ADHD group was more diffuse and extended across motor, attentional and associative regions, whereas children with isolated DCD or ADHD showed more circumscribed and domain-specific patterns of cortical alteration. These findings support the notion that comorbidity is associated with a distinct neurodevelopmental profile rather than a simple overlap of the two disorders (Langevin et al., 2015). Using diffusion tensor imaging, the same authors further demonstrated disorder-specific patterns of white matter microstructural alterations, with frontal abnormalities characterizing ADHD, motor and somatosensory pathways characterizing DCD, and combined frontal–parietal corpus callosum alterations observed in DCD + ADHD (Langevin et al., 2014). In line with these findings, a large structural MRI study on 226 children adopting both dimensional and categorical approaches showed that motor coordination abilities, particularly aiming and catching, were significantly associated with volumes of the premotor and motor cortex and of the superior cerebellar lobules. Importantly, these structure–motor relationships were not moderated by the severity of ADHD symptoms. In the categorical level, children with DCD showed reduced volumes in these regions compared with typically developing peers; no significant differences emerged between children with DCD alone and those with DCD and comorbid ADHD, suggesting that the neural substrates of basic motor coordination are largely shared across DCD with and without ADHD, rather than being specifically altered by comorbidity (Shaw et al., 2016).

Adopting a transdiagnostic approach, Bonthrone et al. (2024) combined neuropsychological assessment and structural MRI in children with DCD and ADHD showing that attentional and executive difficulties were highly prevalent and largely independent of motor impairment severity. At the neuroanatomical level, motor skills were associated with reduced surface area of the left posterior cingulate cortex and altered corticospinal tract morphology, while inattentive symptoms were related to increased cortical thickness in posterior cingulate regions, suggesting partially dissociable structural correlates of motor and attentional difficulties in DCD.

Overall, structural MRI studies indicate that gross volumetric measures alone may be insufficient to consistently capture neural correlates of DCD. Indeed, studies focusing on cortical, cerebellar and subcortical volumes have reported subtle or non-significant group differences, including inconsistent findings at the cerebellar level (Grohs et al., 2021; Fernandez et al., 2022). These results suggest that cerebellar involvement in DCD may not be reliably detectable using macrostructural measures and may instead be better characterized through functional and network-level analyses.

In line with this view, task-based fMRI studies have provided clearer evidence of disorder-specific functional profiles, particularly when directly comparing DCD and ASD. Butera et al. (2025) reported reduced activation of cerebellar regions, in children with DCD during motor execution and imitation tasks, whereas children with ASD showed a distinct pattern of neural activation. Importantly, cerebellar activation was significantly associated with kinematic measures of movement control, supporting a specific role of cerebellar dysfunction in DCD.

Diffusion MRI studies suggest that white matter alterations in DCD are subtle and primarily involve sensorimotor association pathways. Using advanced tractography techniques, Kilroy et al. (2022a, 2022b) reported disorder-specific white matter signatures, with alterations in motor-related tracts in DCD and fronto-parietal pathways in ASD. Importantly, Hyde et al. (2019) demonstrated that these alterations may remain undetected when using conventional DTI but become evident with higher-order diffusion models such as constrained spherical deconvolution, which revealed reduced apparent fiber density in the left superior longitudinal fasciculus in individuals with DCD, associated with poorer motor performance Further evidence for altered large-scale brain organization in DCD comes from resting-state fMRI studies. McLeod et al. (2014, 2016) demonstrated reduced functional connectivity within motor networks in children with DCD, ADHD and DCD + ADHD, with disorder-specific patterns involving basal ganglia, thalamic, cerebellar and cortical regions. In particular, children with DCD showed reduced connectivity between the primary motor cortex and basal ganglia, while children with DCD + ADHD exhibited additional alterations involving higher-order visuospatial regions.

More recent whole-brain resting-state analyses have reinforced these findings. Rinat et al. (2020) reported reduced functional connectivity between the sensorimotor network and posterior cingulate cortex, precuneus and posterior temporal regions in children with DCD and associated ADHD, suggesting impaired integration between motor, attentional and default-mode networks. Extending this work, Nemmi et al. (2023), in a large pediatric sample including children with DCD, developmental dyslexia and their comorbidity, showed that resting-state functional measures were more informative than structural measures alone in distinguishing clinical groups. In particular, functional networks involving cerebellar and frontal regions contributed most to group differentiation, and children with comorbid DCD + developmental dyslexia exhibited a distinct pattern of brain functioning compared with children with isolated disorders.

Only few task-based fMRI studies focusing on executive control further highlight the impact of comorbidity. Thornton et al. (2018) showed that reduced activation during motor inhibition tasks was observed only in children with DCD + ADHD, particularly in precentral and frontal regions, supporting the notion that comorbid conditions are associated with more severe and widespread functional alterations. Finally, a study using SPECT conducted on 10 drug-naïve adolescents with ADHD without DCD and 5 adolescents with ADHD comorbid DCD, showed lower regional cerebral blood flow (rCBF) of bilateral frontal lobe, inferior parental lobe, and increased rCBF of right posterior cingulate gyrus, anterior lobe of cerebellum in ADHD and DCD group compared to ADHD without DCD; in addiction decreased rCBF in the right occipital, inferior temporal lobe was found in ADHD comorbid DCD group after Methilphenidate (Yeh et al., 2012).

#### 3.2.2 EEG

There are few reports regarding the relationship between DCD associated to ASD or ADHD. In one of the studies reviewed, the relationship between motor difficulties and attentional deficits, measured through EEG, was examined among children with DCD without ADHD comorbidity (*n =* 57), children with DCD + ADHD (*n =* 29), and typically developing controls (*n =* 99). The results showed that children with DCD (both with and without ADHD) had significantly lower scores in the Movement ABC test, although children with pure DCD displayed more pronounced ball skill difficulties compared both to controls and to their peers with DCD + ADHD. With regard to the EEG-based attention index, both children with DCD alone and those with DCD + ADHD scored lower than controls, but no significant differences emerged between the two clinical subgroups. Regression analyses indicated that the attentional index accounted for a relevant proportion of the variance in motor performance in both groups: about 14% in children with DCD and 17% in those with DCD + ADHD (Fong et al., 2016a).

#### 3.2.3 Eye tracking and oculomotor behavior

Ocular movement abnormalities have been investigated in children with DCD and other neurodevelopmental comorbidities in few studies only.

In studies comparing ocular abilities in children with DCD and DD, assessed through the Developmental Eye Movement test (DEM) it was found that children with isolated DCD mainly showed problems with accuracy, whereas children with DD displayed more pronounced difficulties in terms of execution speed and number of errors. In children with comorbid DCD + DD, a more severe impairment was observed, with a combined compromise of speed, accuracy, and oculomotor parameters, highlighting an additive effect of the two disorders (Bellocchi et al., 2021). Eye tracking was also used in a study aimed at examining visual attention to socially relevant stimuli (faces, eyes, social scenes) in children with ASD, DCD, and TDC. The study found that children with DCD spent less time fixating on faces and the eye region compared to TDC, but more than children with ASD, thus placing themselves in an intermediate position. Moreover, both DCD and ASD children showed a reduced ability in gaze following (shifting gaze from the eyes/face to the object being observed), suggesting a lower spontaneous sensitivity to social signals conveyed through gaze (Sumner et al., 2018b).

#### 3.2.4 Gait analysis in in DCD with other neurodevelopmental comorbidities

No studies on gait analysis in children with DCD and other neurodevelopmental comorbidities were reported.

## 4 Discussion

In this work, we analyzed studies on the neurophysiological and neurobiological correlates of children with DCD, using different methodologies such as EEG, functional neuroimaging (fNIRS, fMRI and SPECT), eye-tracking and gait analysis, and relating these data to the main neurodevelopmental comorbidities (ADHD, ASD, dyslexia). Overall, the studies demonstrate structural, functional, and connectivity alterations in several brain areas in children with DCD, both in the isolated form and in the presence of comorbidities, compared with healthy controls. Despite the heterogeneity of the techniques employed, a coherent pattern emerges that allows DCD to be interpreted not merely as a motor deficit, but as a disorder involving the quality of the connections between sensory, motor, and executive systems. The consistency of the findings, despite the diversity of instruments used, supports a unified interpretation of DCD, which represents a relevant contribution of this review.

From a functional perspective, the presence of atypical patterns of brain activation and connectivity, with the recruitment of alternative circuits, suggests the hypothesis of reduced automatization of movement by the motor networks involved and an increased reliance on compensatory cognitive strategies, which may negatively influence the fluidity and stability of movements in children with DCD (Zwicker et al., 2012; Debrabant et al., 2013; Kashiwagi et al., 2009; Joshi et al., 2022; Caçola et al., 2018; Huang et al., 2024; Al-Yahya et al., 2023). Importantly, recent fMRI findings indicate that these atypical activation patterns do not necessarily reflect reduced task engagement or impaired performance, but rather a shift toward less efficient and more cognitively demanding neural strategies. In particular, studies employing longitudinal and ecologically valid paradigms show that children with DCD may achieve behavioral performance comparable to typically developing peers, albeit through increased recruitment of frontal and visual regions, suggesting a higher cognitive cost associated with motor execution and visuomotor decision-making (Zwicker et al., 2010; Zwicker et al., 2011; Rapos et al., 2026). DCD is not merely a motor difficulty, but an execution modality that increasingly relies on cognitive control. This dependence on frontal processing, coupled with reduced activation of premotor and cerebellar areas, represents a fragile compensatory mechanism that tends to lose effectiveness in tasks requiring automatization or increased attentional load. This pattern is further supported by fNIRS and task-based fMRI evidence showing asymmetric and inefficient prefrontal recruitment during motor tasks requiring cognitive–motor integration. Increased reliance on left dorsolateral prefrontal regions, together with reduced or absent modulation of right prefrontal areas, suggests a compensatory shift toward explicit control mechanisms, particularly under dual-task or high-demand conditions (Al-Yahya et al., 2023; Joshi et al., 2022).

Studies on MNS, conducted in the context of motor planning and attention during imitation (Reynolds et al., 2015; Reynolds et al., 2019), reinforce the hypothesis that motor difficulties are not limited to execution, but also involve higher cognitive processes, such as planning and inhibitory control, with less efficient reliance on prefrontal networks (Querne et al., 2008; Licari et al., 2015). This interpretation is further supported by recent gait studies showing altered interlimb coordination, increased stride-to-stride variability, and reduced dynamic stability during walking and running, particularly under conditions requiring temporal prediction or sensory integration (Goetschalckx et al., 2024a; Goetschalckx et al., 2024b; Speedtsberg et al., 2018). The involvement of auditory–motor synchronization deficits during paced walking paradigms suggests that impaired temporal processing extends beyond the visuomotor domain, reinforcing the view of DCD as a disorder of predictive control and multisensory integration across motor contexts. This evidence suggests that the cognitive–executive dimension represents a central element in understanding DCD, and that much of its clinical expression arises from the interaction between motor deficits and vulnerabilities in action-control systems.

In addition to functional alterations, some studies show microstructural abnormalities in the white matter, suggesting that motor execution difficulties may also derive from altered sensorimotor integration, attributable to both functional and anatomical dysfunctions (Debrabant et al., 2016; Langevin et al., 2014). Notably, recent structural MRI studies suggest that these brain–behavior relationships are largely dimensional rather than categorical. Associations between gray matter morphology in frontal, parietal and cerebellar regions and motor or motor imagery performance indicate that neural alterations scale with motor proficiency and attentional demands, rather than strictly reflecting diagnostic group membership (Malik et al., 2024; Mukherjee et al., 2025). This further supports a network-based and developmental interpretation of DCD, in which variability in motor outcomes reflects differences in the efficiency and integration of distributed neural systems. In particular, the reduced integrity of fronto-parietal pathways and interhemispheric connections indicates that the disorder involves coordination mechanisms between brain regions rather than abnormalities confined to single areas. Taken together, these findings suggest that motor difficulties in DCD is related to inefficient large-scale network integration and developmental alterations in brain connectivity, reinforcing the conceptualization of DCD as a disorder of distributed neural systems.

Thanks to electrophysiological studies, reduced interhemispheric integration has also been documented in children with DCD (Blais et al., 2018), indicating that motor deficits may depend not only on functional or structural alterations of individual brain regions, but also on compromised connectivity between different brain systems (Debrabant et al., 2016; McLeod et al., 2014; McLeod et al., 2016). This evidence provides further support for the hypothesis of an integration deficit, which limits the stabilization of motor learning and contributes to the execution variability frequently described in the literature. In line with this interpretation, several EEG studies have shown atypical oscillatory activity, altered functional connectivity, and reduced efficiency of neurocognitive processes supporting motor planning, visuospatial attention, and sensorimotor integration in DCD (Lum et al., 2025; De Castelnau et al., 2008; Pangelinan et al., 2013; Tsai et al., 2009; Wang et al., 2015). Consistently, time–frequency EEG studies during visuospatial working memory tasks have shown that children with DCD exhibit reduced modulation of frontal theta and posterior alpha oscillations during encoding and maintenance phases, together with diminished prestimulus alpha activity associated with poorer task performance (Wang et al., 2017). These findings suggest impaired neural mechanisms supporting attentional allocation and maintenance of task-relevant information, further reinforcing the role of executive–attentional dysfunctions in the motor difficulties observed in DCD. Recent EEG findings further refine this picture by showing that electrophysiological alterations in DCD are task- and frequency-specific, affecting both oscillatory and event-related markers of motor control and learning. In particular, atypical beta modulation during procedural sequence learning and reduced functional connectivity across frequency bands suggest inefficient recruitment of motor networks during skill acquisition (Lum et al., 2025; Song et al., 2025). Moreover, reduced ERN amplitudes during visuomotor adaptation indicate diminished sensitivity to sensory prediction errors and suboptimal updating of internal models, despite partial behavioral compensation with practice (Warlop et al., 2025). Together, these findings support the interpretation that motor learning difficulties in DCD arise from noisy sensorimotor processing and impaired network-level integration rather than from a primary deficit in learning capacity. Moreover, findings of reduced mu desynchronization and diminished error-monitoring responses further indicate impaired neural specialization within the mirror neuron system and suboptimal updating of internal models (Licari et al., 2015; Lust et al., 2019; Warlop et al., 2025), reinforcing the notion that DCD reflects a broader dysfunction in the coordination and integration of distributed neural networks.

The hypothesis that motor difficulties in DCD do not derive exclusively from a primary motor deficit, but also from difficulty in integrating multisensory inputs in an automated manner, is also supported by studies on eye movements (Gomez et al., 2017; Sumner et al., 2018a; Licari et al., 2018; Parr et al., 2020; Bellocchi et al., 2021) and gait analysis (Chia et al., 2013; Deconinck et al., 2006). These studies show, in fact, a less efficient use of visual information to guide movement, with repercussions on the fluidity, precision, and stability of motor execution. The increased reliance on visual guidance represents an additional behavioral indicator of the difficulty in constructing automated and stable motor representations. Moreover, evidence from Quiet Eye–based (QET) interventions indicates that atypical gaze strategies in DCD are not merely epiphenomenal but reflect core deficits in anticipatory control mechanisms; improvements observed after QET (Miles et al., 2015; Wood et al., 2017) underscore the malleability of visuomotor planning and suggest that impaired predictive control contributes meaningfully to DCD symptomatology further supporting the hypothesis of disrupted anticipatory and multisensory integration processes.

With regard to comorbidities, most of the studies analyzed concern the association between DCD and ADHD, which represents the most frequent co-occurrence, although we also examined a smaller number of studies concerning ASD and DD. All studies agree that comorbidities are associated with a worsening of the neurobiological profile, although not in a uniform manner: some evidence supports the hypothesis of an additive effect, in which the specific deficits of each disorder (frontal in ADHD, sensorimotor in DCD) sum up, generating a broader impairment (Langevin et al., 2014), while other studies suggest that comorbidity may configure a distinct neurobiological phenotype, with its own brain developmental trajectories that cannot be reduced to the mere union of deficits (Albajara Sáenz et al., 2021). In both cases, the emerging picture is one of greater vulnerability of the circuits integrating motor control, attention, and executive functions (Albajara Sáenz et al., 2021; Langevin et al., 2014). Furthermore, eye-tracking studies comparing DCD + ASD and DCD + DD groups (Bellocchi et al., 2021; Sumner et al., 2018b) indicate that comorbidity amplifies visuomotor and attentional abnormalities, supporting the interpretation that multisensory integration deficits become more pronounced when additional neurodevelopmental conditions are present. The observation of specific alterations in social gaze allocation and gaze-following (Sumner et al., 2018b) further suggests that comorbidity may introduce qualitatively different neurocognitive challenges, rather than simply exacerbating existing DCD characteristics. Consistently with what has been observed in pure DCD, studies on comorbidities have also reported structural, functional, and connectivity alterations, confirming the hypothesis that the co-occurrence of neurodevelopmental disorders is associated with greater complexity and severity of the neurobiological profile (Albajara Sáenz et al., 2021; Langevin et al., 2014; McLeod et al., 2014; McLeod et al., 2016). Overall, the available evidence converges toward a unified interpretative model in which DCD can be conceptualized as a disorder of connectivity and multisensory integration. This framework provides a useful theoretical lens through which to reinterpret the wide variability of clinical and instrumental findings reported in the literature.

It is also important to acknowledge the limitations of the existing literature: many studies include small sample sizes, heterogeneous experimental paradigms, and cross-sectional designs that do not allow for an accurate evaluation of the developmental trajectories of the networks involved. These limitations should be taken into account when interpreting the results and highlight the need for multimodal longitudinal studies in larger cohorts in order to confirm the same findings especially in EEG and neuroimaging sections. In addition, regarding the comorbidities associated with DCD as well as ASD and ADHD, transdiagnostic/shared network models should be considered.

Finally, this integrative perspective has relevant clinical implications, suggesting the utility of assessments that combine motor, cognitive, attentional, and sensory indicators, as well as rehabilitation approaches that target not only motor abilities but also multisensory integration processes and executive functions.

## 5 Conclusion

In conclusion, the scientific evidence reviewed indicates that DCD cannot be considered a purely motor disorder, but rather a complex neurodevelopmental condition characterized by widespread structural, functional, and connectivity alterations. It is important to highlight that the disorder is still frequently underdiagnosed, partly because it often occurs in comorbidity with other neurodevelopmental disorders and is overshadowed by diagnoses perceived as more impactful on global functioning, such as ADHD or ASD. However, the findings of this review show that comorbidity represents a significant factor of severity, further complicating and reducing the effectiveness of rehabilitative interventions, thus contributing to the persistence of difficulties over time. Moreover, the underestimation of DCD and the consequent lack of targeted interventions are associated with a considerable impact on the child’s mental health, with an increased risk of internalizing disorders (anxiety, depression) and social withdrawal (including avoidance of sports activities and bullying).

These findings also underscore the need to systematically include DCD screening in the evaluation of children, particularly those with neurodevelopmental disorders, and to develop multimodal approaches integrating neuroimaging, neuropsychological data, and clinical observation. Such strategies could support the development of personalized therapeutic interventions, ultimately improving long-term outcomes. However, future research priorities, such as longitudinal designs, multimodal integration at the individual level, and implications for personalized intervention strategies have to be considered.

## Glossary

ACC: Anterior Cingulate Cortex
ADHD: Attention-Deficit/Hyperactivity Disorder
ASD: Autism Spectrum Disorder
BOLD: blood-oxygen-level-dependent
DCD: Developmental Coordination Disorder
DD: dyslexia
DEM: Developmental Eye Movement test
DLPFC: dorsolateral prefrontal cortex
DTI: Diffusion Tensor Imaging
EEG: Electroencephalography
FA: Fractional Anisotropy
fNIRS: functional Near-Infrared Spectroscopy
fMRI: Functional Magnetic Resonance Imaging
ICD: International Classification of Diseases
IFG: inferior frontal gyrus
IPC: Inferior Parietal Cortex
M-ABC: Movement Assessment Battery for Children
MFC: Medial Frontal Cortex
MNS: mirror neuron system
MRI: Functional Magnetic Resonance Imaging
QoL: Quality of Life
RD: Radial Diffusivity
SMA: supplementary motor area
SM1: primary sensorimotor cortex
SPECT: Single-Photon Emission Computed Tomography
TDC: typically developing controls
