# Supplementary material for: Advancing understanding of developmental coordination disorder in children: data from the literature
Source: Front Hum Neurosci. 2026 Jun 3;20:1779776. doi: 10.3389/fnhum.2026.1779776 (PMC13272446; doi:10.3389/fnhum.2026.1779776)
Supplement: Supplementary file 1 [file Table_1.docx]

| Study, Author, Year | Design | Population | | | Age | Assessment for  DCD/Motor Performance and comorbidity | Analyzing methods | Main Findings | Limitations |
| --- | --- | --- | --- | --- | --- | --- | --- | --- | --- |
| Debrabant et al., 2013 | Cross-sectional observational study | | DCD: 17  TDC: 17 | | 7-10 years | DCD:  -MABC-2 ( ≤ the 5^th^ percentile for DCD)  -Estimated total IQ score of 85  -No other diagnosed developmental disorders | *fMRI:* motor reaction to sequences of visual stimuli with predictive or unpredictive interstimulus intervals (ISIs) | **DCD**: ↓ reaction time advantage to predictive ISIs  **TDC**: ↑ activation in DLPFC and IFG for responses at  unpredictive as opposed to predictive ISIs  DCD: ↓ activation in DLPFC, left posterior cerebellum and right temporo-parietal junction (TPJ) in the same contrast; activation of TPJ is associated with RT | Lack of utilization of dual task paradigms to provide more reliance on sensory feedback processing;  Small sample |
| Joshi et al., 2022 | Experimental study (fNIRS) | | DCD: 48 TDC: 37 | | 13.9±0.3 yrs | -Hand–eye coordination task  -Behavioral measures | fNIRS: cortical oxygenation in prefrontal cortex (PFC); analysis of single- vs dual-task conditions | **DCD:** poorer cognitive performance in dual-task; fNIRS: ↓ PFC activation (esp. right frontal) and reduced neural efficiency.  **DCD + TD:** motor performance worsened in dual-task (no group difference). | Pilot study, not generalizable;  limited to a single motor coordination task. |
| Huang et al., 2024 | Cross-sectional experimental study | | DCD: 20 TDC: 20 | | DCD: 9.88 ± 1.67;  TDC: 9.87 ± 1.59 | Double-step reaching task | fNIRS: 10 ROIs: bilateral primary motor, primary somatosensory, premotor, superior/inferior parietal cortices (SPC-IPC).  ΔHbO as cortical activation index | **DCD:** longer correction times (MTdiff); fNIRS: no significant SPC activation in jump condition; ΔHbO in left SPC correlated negatively with MTdiff (r = −0.560)  **DCD + TDC:** SPC and IPC showed main effects of condition (jump > no-jump); MTdiff negatively correlated with SPC activation (r = −0.598). | fNIRS cannot detect cerebellar activity;  short test duration with unbalanced trial ratio; additional task actions may have influenced results |
| Al-Yahya et al., 2023 | Non-randomized interventional study | | DCD: 46 TDC: 38 | | Adolescents | 7-week stepping training (single- and dual-task);  behavioral performance | -fNIRS: PFC activation during stepping, Stroop, dual-task  -fMRI: baseline structural and functional activation during stepping (sensorimotor cortex, cerebellum) | **DCD:** at follow-up, Stroop dual-task errors remained higher vs single-task;  fNIRS: less efficient DLPFC recruitment  **DCD + TD**: step variability ↓ and frequency ↑ after training; fMRI: sensorimotor and cerebellar activation during stepping (no group/time effects); greater fronto-parietal activation correlated with lower variability | Non-randomized;  attrition at follow-up;  no significant structural/functional MRI training effects after 7 weeks (possibly due to short intervention). |
| Debrabant et al., 2016 | Cross-sectional observational study | | DCD: 21  TDC: 20 | | 8–10 yrs | MABC-2 (≤5th percentile for DCD, >16th percentile for TDC);  VMI | 3T MRI: DTI metrics (FA, RD, AD); deterministic tractography of sensorimotor tracts; graph theory (clustering coefficient, global/local efficiency, small-worldness); regression and discriminant analyses | **DCD:** poorer visuomotor performance (Beery VMI);  ↓ FA and ↑ RD in left internal capsule; ↓ clustering coefficient and global efficiency; reduced nodal efficiency in cerebellum (lobules IV–VI), left cingulate, right superior parietal gyrus. In DCD, global efficiency correlated with visuomotor performance.  **DCD + TD:** FA reduction correlated with VMI trace performance. Discriminant model (cerebellum VI + right superior parietal) classified groups with 87.8% accuracy. | Small sample;  deterministic tractography less sensitive than probabilistic models;  edge weighting not standardized;  VMI assesses outcomes but not underlying processes. |
| Caçola et al., 2018 | Preliminary experimental study | | DCD: 6 TDC: 6 | | 8–12 yrs | MABC-2,  Beery-VMI,  ETCH (handwriting) fine-motor tasks | fNIRS: ΔHbO/ΔHbR changes;  GLM analysis; a  ctivation maps per task and group | **DCD:** poorer fine-motor, visuomotor, and handwriting scores; atypical cortical activation with more diffuse/ipsilateral recruitment and less premotor involvement (suggesting less efficient strategies).  **TDC:** typical contralateral or focal activation patterns depending on task. | Very small sample (N=12); preliminary, not generalizable;  fNIRS cannot detect cerebellar/deep structures; no real-time motor performance recording. |
| Reynolds et al., 2015 | Cross-sectional observational study | | DCD: 14  TDC: 12  Only males | | 8-12 years | DCD:  -movement difficulties interfering with  daily living evidenced through referral to our programs; MABC-2 scores ≤16th percentile  -excluded other neurodevelopmental disorders  Other test:  -Edinburgh Handedness Inventory (EHI; Oldfield, 1971), only right-handers (score ≥ 40) were included  - Postural Praxis imitation test of the Sensory Integration and Praxis Tests (SIPT) to evaluate imitative ability  BEHAVIORAL TASKS:  finger sequencing task with dominant (right) hand:  -action observation,  -action execution  -action imitation; | Mirror neuron system (MNS) fMRI: Cortical activations of mirror neuron regions, including IFG, ventral premotor cortex, anterior inferior parietal lobule and superior temporal sulcus | DCD: ↓ cortical activation mirror neuron regions: precentral gyrus and IFG, as well as in the posterior cingulate and precuneus complex when observing the sequencing task;  ↓ activation pars opercularis, a primary MNS region, during  imitation | -No quantitative performance measures were collected;  -previous practise task: memory and propioceptive feedback;  -not all children in the DCD  group had a formal diagnosis of DCD, and inclusion was based on MABC-2 scores. |
| Rapos et al., 2026 | Cross-sectional observational **case–control study** | | DCD: 24  (N.B. In DCD group were included participants with and without co-occurring ADHD; the exact number of DCD + ADHD cases was not reported”.  TD: 24 | | 7-12 years | DCD: DSM-5 criteria, MABC-2**,** Developmental Coordination Disorder Questionnaire (DCDQ; parent report**)**. Cognitive ability: Kaufman Brief Intelligence Test – 2nd edition **(KBIT-2)**, visual perceptual skills with the Test of Visual Perceptual Skills – 4th edition **(TVPS-4)**. **Conners 3rd Edition ADHD Index (parent report)**. | **fMRI** data were acquired during a **virtual decision-making task** (motor planning, action selection and execution).  Brain activity was measured using **task-based BOLD (blood-oxygen-level-dependent) signal changes** 🡪 identify group differences in neural networks involved in motor decision-making. | DCD: reduced and/or atypical BOLD responses in cortical regions involved in motor planning, action selection, and executive control, indicating less efficient neural recruitment during decision-making in action contexts + poorer motor and task performance.  N.B: although children with co-occurring ADHD were included in the DCD group, no subgroup analyses were performed 🡪 the specific contribution of ADHD comorbidity to the observed activation patterns could not be determined. | **Cross-sectional design**; **Inclusion of children with co-occurring ADHD** without reporting their exact number;  **Relatively modest sample size**.  **fMRI limitations:** high sensitivity to head motion and indirect measurement of neural activity via BOLD signal changes. |
| Mukherjee et al., 2025 | Cross-sectional structural MRI study investigating associations between gray matter volume and motor imagery performance. | | DCD: 15  TD: 31 | | 8-14 years | **DCD**: DSM-5 criteria  **Motor imagery performance**: Hand Rotation Task (HRT) 🡪 participants judged left vs right hands at various angles, with *response time (RT)* and *accuracy* recorded. Mean inverse efficiency scores (IES) calculated; lower IES = better performance.  **Attentional difficulties**: ADHD Rating Scale-IV. | **Structural MRI** acquired at **3T**.  **Voxel-based morphometry (VBM)** 🡪 quantify regional gray matter volume.  Correlations between gray matter volume and motor imagery performance were examined to identify brain regions associated with motor planning and representation. | DCD: ↓ **gray matter volume in motor-related regions** including precentral and postcentral gyri.  All participants: **better motor performance** 🡪 greater gray matter volume in **sensorimotor cortices** (all participants). | **Cross-sectional design;**  **Sample size;**  **Behavioral assessment limitation:** motor imagery tasks may not fully capture real-life motor planning abilities.  **Structural MRI limitation:** VBM measures volume but does not assess connectivity or functional activation. |
| Bianco et al., 2024 | Cross-sectional observational study; combined behavioral and structural MRI | | DCD: 19  TD: 40 | | 6-14 years | **DCD**: DSM 5 criteria; Bruininks–Oseretsky Test of Motor Proficiency, 2nd edition, short form (**BOT-2 SF**); (**DCD-Q**),  Inattention/ADHD symptoms: **ADHD Rating Scale-IV;**  Schematic overview of the serial reaction time **(SRT)** task | **Behavioral**: SRT RT and rebound effect for procedural learning;  **MRI**: T1-weighted structural images; cerebellum, basal ganglia, and frontal volumes extracted via FreeSurfer; **Correlation analyses** between regional volumes and SRT performance controlling for inattention. | DCD: atypical procedural learning on the SRT task compared to TD children.  DCD: did not show RT improvement across sequence blocks.  Group differences persisted after controlling for inattention/ADHD symptoms.  DCD: ↓ cerebellar volume was associated with task performance. | Small MRI sample (n=10 DCD)  Cross-sectional design limits causal inference Trend-level group differences in cerebellar volume  Not formal ADHD diagnosis  Structural MRI does not capture functional connectivity. |
| Rinat et al., 2020 | Cross-sectional resting-state functional MRI (rs-fMRI) case–control study | | DCD: 35  TD: 23 | | 8-12 years | **DCD:** standardized clinical criteria (DSM-5) and motor assessment (MABC-2).  **TD:** No motor or developmental disorders.  **Resting-state fMRI:** Assessed whole-brain functional connectivity | Resting-state functional MRI (rs-fMRI) acquired at 3T.  Independent Component Analysis (**ICA**) used to identify 19 resting-state networks.  Between-group comparisons of functional connectivity between children with DCD and TD controls, controlling for age and sex. | DCD: altered functional connectivity between the sensorimotor network and posterior cingulate cortex (PCC), precuneus, posterior middle temporal gyrus (pMTG) | Cross-sectional design;  Functional connectivity differences may be influenced by unmeasured behavioral or attentional variables.  Resting-state analyses cannot determine directionality of network disruption.  Potential motion artifacts in pediatric rs-fMRI design. |
| Le et al., 2021 | Cross-sectional behavioral + structural MRI study examining synchronization, procedural learning, and retention of temporal sequences. | | DCD: 11  TD: 19 | | 6-14 years | **DCD:** Standardized clinical criteria and motor assessment (MABC-2);  **Procedural learning:** synchronization to regular auditory/visual temporal sequences;  **Verbal timing task;**  **Neuroimaging:** MRI to measure cortical thickness in sensorimotor, frontal, and parietal regions. | Behavioral performance on synchronization and procedural learning tasks compared between DCD and TD.  Structural MRI used to measure cortical thickness in a priori regions (sensorimotor, frontal, parietal).  Correlations between task performance and cortical thickness examined. | DCD: general deficit in synchronizing regular temporal verbal sequences, regardless of sensory modality (auditory or visual).  DCD: specific deficit in learning and retention of auditory non-regular temporal sequences compared to TD. | Moderate sample size;  Cross-sectional design;  Exact ADHD/attentional comorbidity details are not fully reported;  Cortical thickness measures do not directly assess neurofunctional mechanisms. |
| Hyde et al., 2019 | Cross-sectional pilot study | | DCD: 7  TD: 12  N.B.: no participants had ADHD or other neurodevelopmental disorders | | 18-46 years | **DCD:** DSM IV criteria; motor impairment confirmed using:  BOT-2 (≤16th percentile), Adult DCD/Dyspraxia Checklist (ADC) for functional impact and childhood onset. | **Diffusion MRI**: comparison of traditional DTI metrics (FA, MD).  **Constrained Spherical Deconvolution (CSD) metrics**:  Fiber Density (FD)  Fiber Cross-section (FC)  Fiber Density and Cross-section (FDC)  Correlations between white matter metrics and motor performance | Traditional DTI metrics (FA, MD): no significant group differences between DCD and controls  CSD / Fixel-based analysis: DCD: ↓ fiber density and/or cross-section in adults with DCD in several motor-related white matter tracts.  Associations with motor performance: ↓ white matter organization 🡪 poorer motor skills  Demonstration that CSD is more sensitive than standard DTI in detecting white matter abnormalities in DCD.  Motor impairments in DCD may be linked to complex fibre architecture changes, not captured by tensor-based models. | Small sample size;  Adult-only sample:  Limits generalizability to children or adolescents with DCD;  Cross-sectional design;  No ADHD comorbidity included: this increases specificity but limits applicability to the DCD population with comorbidity ADHD  Motor assessment in controls incomplete: not all controls completed BOT-2 testing  Larger, longitudinal studies are needed to confirm results and assess developmental changes. |
| Zwicker et al., 2011 | Cross-sectional case–control fMRI design | | DCD: 7  TD: 8 | | 8-12 years | **DCD:** clinical diagnosys; no neurological disorders, intellectual disability, or contraindications for MRI  **TD:** no history of motor, neurological, or developmental disorders | **Motor task**: a visuomotor tracking (to control a joystick to follow a moving target on a screen).  **MRI acquisition**: blood-oxygen-level-dependent (**BOLD**) fMRI  **Whole-brain analysis**: comparison of activation during motor task performance between groups | *Behavioral Results:*  **DCD:** poorer motor performance;  Both groups improved with practice.  *fMRI Results:*  DCD: ↓ activation in cerebellum, basal ganglia, parietal regions involved in sensorimotor integration;  ↑ activation in prefrontal cortex, anterior cingulate cortex. | Small sample size;  Cross-sectional design;  fMRI motion artifacts;  Task-specific findings: may not generalize to real-world motor skills;  ADHD symptoms not formally controlled (this may confound results). |
| Zwicker et al., 2010 | Cross-sectional case–control fMRI study | | DCD: 10  TD: 8 | | 8-12 years | **DCD**: DSM-IV criteria; motor performance below age norms; motor difficulties interfering with daily activities.  **TD**: no history of motor or neurodevelopmental disorders | Motor Task: hand-held joystick to control a cursor, requiring goal-directed fine motor movements. Repeated blocks to allow motor learning  **MRI acquisition (fMRI)**: blood-oxygen-level-dependent (BOLD) imaging during task performance   - Analyses focused on **task-related brain activation differences** between groups. | *Behavioral Results:*  **DCD:** poorer motor performance; motor learning evident but less efficient in DCD group.  *fMRI Results:*  DCD: ↓ activation in cerebellum, basal ganglia, parietal regions;  ↑ activation in prefrontal cortex, anterior cingulate cortex 🡪 children with DCD use cognitive control networks to compensate for deficits in automatic motor networks | Small sample size;  Cross-sectional design;  Pediatric fMRI susceptible to motion artifacts;  Findings may not generalize beyond the specific task used;  ADHD or attention-related symptoms not formally controlled. |
| Querne et al., 2008 | Cross‑sectional functional MRI (fMRI) study | | DCD: 9  TD: 10 | | 8-13 years | **DCD:** clinical diagnosis | **Task‑related fMRI acquisition** during go–nogo performance.  **Behavioral analysis**: Evaluation of correct inhibitions, response time, variability.  **Structural Equation Modeling (SEM)**: connectivity between key neural regions involved in attention and inhibition | *Behavioral Results*: DCD no significant differences in correct inhibitions;  *Connectivity differences*:  DCD: ↑ path coefficients from both MFC and ACC to IPC, especially in the left hemisphere; ↓ path coefficients between striatum and parietal cortex. Children with DCD may compensate for inefficient automatic inhibition by engagement of ACC, suggesting top‑down control reliance during inhibition tasks. | Small sample size;  Cross‑sectional design;  Behavioral measures showed slower responses but not failure in inhibition: this complicates the interpretation of results;  ADHD symptomatology was not explicitly controlled or measured;  SEM assume predefined network structure and may not capture whole‑brain dynamics. |
| Brown-Lum et al., 2020 | Cross-sectional neuroimaging study using diffusion tensor imaging (DTI) metrics | | DCD: 31  TD: 31 | | 7-12 years  Mean age: 9.7 years | **DCD**: DSM-5 criteria; MABC-2 (score ≤16th percentile);  **TD**: MABC-2 scores ≥25th percentile and no history of motor difficulties  **Attention**: Conners 3 ADHD Index for parent-reported inattention/hyperactivity  **DTI metrics** **analyzed**: Fractional Anisotropy (FA) – directional;coherence of white matter; Mean Diffusivity (MD) – overall water diffusion; Radial Diffusivity (RD) – myelination Indicator; Axial Diffusivity (AD) – axonal integrity indicator | **MRI acquisition:** 3T scanner with diffusion-weighted sequences;  **Tract-based spatial statistics (TBSS):** voxelwise comparison of FA, MD, RD, AD between DCD and TD groups; | *Global white matter differences*:  DCD: ↓ FA in widespread white matter tracts (corpus callosum, corticospinal tracts, superior longitudinal fasciculus) corresponding ↑ MD and RD.  Microstructural differences remained significant after controlling for ADHD symptom scores 🡪 DCD-related white matter alterations are not fully explained by attentional difficulties. | Cross-sectional design;  DTI limitations: cannot directly measure fiber density or crossing fibers, potential partial volume effects;  Small sample size;  Age range: findings may not generalize to older or younger children. |
| Reynolds et al., 2019 | Cross-sectional case–control fMRI study | | DCD: 10  TD: 9 | | 8.25-12.75 years | **DCD:** DSM-5 criteria; MABC-2 ≤16th percentile.  **ADHD:** symptoms severity was measured and statistically controlled (parent-reported ADHD symptom questionnaires), not treated as a separate group.  ***Exclusion criteria*:**  Neurological disorders; autism spectrum disorder; intellectual disability; MRI contraindications. | **Experimental Task:** action observation paradigm designed to activate the mirror neuron system  **MRI acquisition;**  Whole-brain voxelwise analyses  **Region-of-interest (ROI) analyses:** inferior frontal gyrus (IFG), inferior parietal lobule (IPL), premotor cortex | **TD**: ↑ activation of the mirror neuron system during action observation, including: *inferior frontal gyrus, inferior parietal cortex, premotor regions*  DCD: ↓ activation in key MNS regions, particularly: *inferior parietal lobule, premotor cortex* | Small sample size;  Cross-sectional design;  ADHD comorbidity: although statistically controlled, cannot be fully disentangled from DCD-related effects;  Task specificity;  Motion sensitivity: pediatric fMRI inherently vulnerable to movement artefacts. |
| Reynolds et al, 2017 | Cross-sectional observational study,  High-resolution MRI | | DCD: 22  TD: 22 | | 7.8–12 years | DCD: ≤16th percentile on MABC-2  TD: ≥20th percentile on MABC-2 | MRI: high-resolution scan (T1-weighted 3D FFE 160 slices  0.575 × 0.575 × 1 mm), voxel-based morphometry (VBM) | DCD: large, right lateralised ↓ in grey matter volume in the medial and middle frontal, and superior frontal gyri compared to controls | Small sample  VBM: no information relating to WM microstructure or tract integrity. |
| Mariën P et al., 2010 | Observational case-control study using functional SPECT imaging | | DCD: 1 | | A single 19‑year‑old male patient | DCD: clinical confirmation Visuomotor integration and executive function;  SPECT imaging | **SPECT functional perfusion imaging** to assess cerebello-cerebral perfusion and functional connectivity | **DCD**:↓ cerebellar perfusion and functional suppression in supratentorial regions involved in planned actions, visuospatial processing, and affective regulation. | Single case study: results are not generalizable;  No control group for comparison;  Limited details on neurocognitive assessments;  Findings are preliminary, requiring confirmation in larger cohorts. |
| Malik et al., 2024 | Cross-sectional MRIstructural MRI study | | 8-12 years | | DCD: 15  DCD + ADHD: 15  TD: 12 | DCD: MABC-2 (motor ability), DCDQ (motor function);  ADHD: Conners 3 ADHD Index | **MRI:** high-resolution T1-weighted scans for **voxel-based morphometry (VBM)** to assess cortical grey matter volume.  **VBM analysis** to compare cortical grey matter volumes between DCD and TD children. | DCD: greater grey matter volume (specifically in the left superior frontal gyrus).   - Lower MABC-2 scores (poorer motor skills) were significantly correlated with greater grey matter volume in the left superior frontal gyrus, left frontal pole, and right middle frontal gyrus. - Higher Conners 3 ADHD Index scores (more attentional difficulties) were associated with greater grey matter volume in several left-hemisphere regions, including superior frontal gyrus, superior parietal lobe, and precuneus.   These findings suggest that increased grey matter volume is linked to poorer motor performance and more attentional problems. | Small sample size and unequal groups (DCD nearly double TD).  DCD and DCD+ADHD combined due to limited sample.  Medication confound: 5/15 children with DCD+ADHD were taking stimulants, potentially affecting results.  Volume-based measures: Grey matter includes both surface area and thickness, which have distinct developmental trajectories.  .  Stratification by age, sex, and medication use is recommended for deeper insights. |
| Albajara Sáenz et al., 2021 | Cross-sectional case-control study | | | DCD + ADHD: 22  DCD + ASD: 16  TD: 17 | 8–12 years | DCDQ-FC; DSM-5 criteria K-SADS-PL  ADI-R  ADOS  CARS | **Structural MRI** at 3T was acquired and analyzed using voxel-based morphometry (**VBM**) to measure regional gray matter volume in the pre-central and post-central gyri, inferior parietal cortex, and superior, middle, and medial frontal gyri.  **Correlations between DCDQ-FC scores and gray matter volume** were performed to examine the relationship between motor performance and brain structure | **DCD+ASD**: poorer general coordination (lower DCDQ-FC scores) was associated with reduced gray matter volume in the **right medial frontal gyrus**.  **DCD + ADHD**: greater overall motor impairment correlated with reduced gray matter volume in the **right superior frontal gyrus**.  **All participants**: poorer control during movement was linked to **right superior frontal gyrus** volume, and general coordination was linked to **left inferior parietal cortex volume**. | Small sample  Several scans had to be excluded due to poor quality  High rate of ADHD comorbidity within the ASD group  Cross-sectional design  Use of parent-reported questionnaires (DCDQ) |
| McLeod et al., 2016 | Cross-sectional observational fMRI study | | DCD: 6  ADHD: 19  DCD + ADHD: 14  TD: 21 | | 11-13 years | DSM IV  DICA-IV  CPRS  mABC-2  MAND | Resting-state **fMRI at 3T** was acquired and analyzed to examine **functional connectivity** of the primary and sensory motor cortices (SM1) within and between hemispheres. Correlations between motor performance scores (MABC-2 and MAND) and functional connectivity were performed to examine the relationship between motor abilities and brain network organization. | **All participants:** motor performance scores correlated with connectivity strength between SM1 and subcortical motor regions (basal ganglia and cerebellum), highlighting the relationship between motor abilities and brain network organization.  **DCD/ADHD + DCD:** atypical within- and between-hemisphere functional connectivity of SM1 regions, with poorer motor performance correlated with **altered connectivity to the anterior cingulate cortex (ACC), supplementary motor area, and visuospatial association region**s.  **ADHD + DCD:** altered connectivity of left thalamus and right cerebellum (lobule V), stronger connection with the **left SM1**. Stronger connections of bilateral precuneus, middle frontal gyri, and inferior lateral occipital cortices with the left SM1.  **TD**: the **right motor cortex** showed stronger intra-hemispheric connections with the thalamus and contralateral connections with the cerebellum. | Small sample size.  DCD group met only 3/4 DSM-V criteria for the disorder  The age of onset of motor coordination difficulties was not specified.  All children with ADHD (except one) were taking methylphenidate |
| Thornton et al., 2018 | Observational case-control study with functional imaging (fMRI) | | DCD: 9  ADHD: 20  DCD + ADHD: 18  TD: 20 | | 8-17 yrs | DSM IV  DICA-IV  CPRS  mABC-2  DCDQ-FC  Motor response inhibition task (“Go/No-go”) | Task-based **fMRI** data were analyzed using **voxel-wise comparisons** to identify differences in brain activation between groups during the motor inhibition task. Group contrasts were performed to examine regions involved in motor control and inhibition and to identify patterns specific for each group. | **DCD:** No significant differences in brain activation.  **ADHD:** No significant differences in brain activation.  **DCD + ADHD:** Reduced activation (BOLD response) in the **right precentral and postcentral gyri**, as well as in the **left superior and middle frontal gyri**. Decreased activation was observed in both the **primary motor cortex** and the **primary sensory cortex**.  **All participants:** TD children showed typical activation patterns in motor and sensory regions during the Go/No-go task. | Cross-sectional study: causal relationships cannot be determined.  Focus on a single task.  Analysis limited to primary motor areas. |
| Langevin LM et al., 2014 | Cross-sectional study with diffusion tensor imaging **(DTI)** | | DCD: 9  ADHD: 23  DCD+ADHD: 23  TD: 26 | | 8-17 yrs | DSM-IV  MABC  DCD-Q  DICA-IV  Conners’ Parent Rating Scale–Revised (CPRS-R) | *Neuroimaging*: diffusion Tensor Imaging (**DTI**) at 3T was acquired to assess white matter microstructure.  *DTI metrics*: Fractional Anisotropy (**FA**), Mean Diffusivity (MD), Radial Diffusivity (RD), Axial Diffusivity (AD) in tracts of interest.  *Behavioral measures*: motor and attentional performance scores were correlated with imaging findings | **DCD only**: ↓ FA in the parietal corpus callosum and left superior longitudinal fasciculus, suggesting **impaired interhemispheric and intrahemispheric motor connectivity**.  **ADHD only**: ↓ FA in the frontal corpus callosum, consistent with **alterations in attention and executive control pathways**.  **DCD + ADHD**: extensive white matter alterations across both frontal and parietal regions of the corpus callosum. ↓ FA was significantly associated with deficits in motor coordination and attentional control.  **ALL**: Better motor and attentional scores were positively correlated with FA values in specific tracts 🡪 **higher white matter integrity supports better functional outcomes**. | Cross-sectional study: causal relationships cannot be determined  Small number of children with DCD only.  High heterogeneity in the severity and presentation of motor impairments within the DCD group.  DTI analysis restricted to a limited number of tracts and limited resolutions. |
| Langevin LM et al., 2015 | Cross-sectional observational structural **MRI study** (cortical thickness analysis) | | DCD: 14  ADHD: 10  DCD + ADHD: 10  TD: 14 | | 8-17 yrs | DSM-V  MABC  DCD-Q  MAND  DICA-IV  Conners’ Parent Rating Scale–Revised (CPRS-R)  For *intellectual functioning:* WASI  For *attention and executive functions:* auditory Attention, Response Set, inhibition Switching subtests (NEPSY-II) 🡪 deficits defined as scores < 8. | **MRI** acquisition: structural T1-weighted scans used to compute cortical thickness. | **DCD + ADHD**:   - *Frontal and parietal lobes*: ↓ cortices in the left frontal pole and left superior frontal gyrus. ↓ cortices in the right primary motor cortex and right superior parietal lobule. - *Temporal and occipital lobes*: ↓ cortical thickness in the bilateral temporal poles. - *Correlations with motor and attentional performance*: ↑ cortical thickness in different regions 🡪 better motor and attention task performance.   **DCD only / ADHD only**: cortical thinning was less pronounced and more localized in focal regions.  Typically developing (**TD**). no significant cortical thinning. | Cross-sectional study: causal relationships cannot be determined  Small sample size.  Wide age range: a period of rapid brain development.  Lack of longitudinal data.  Possible influence of unidentified comorbidities (other neuropsychiatric disorders besides DCD and ADHD were not excluded).  Neuroimaging analysis limitations: cortical thickness measures do not provide direct information about functional connectivity between brain regions. |
| Shaw et al, 2016 | Cross-sectional | | 226 children  Only 170 (with completed data) for DSM-5 application:  1.DCD: 22  2.ADHD: 42  3.DCD + ADHD: 41 | | 4 and 16.9 yrs | -DSM-5  -Diagnostic Interview for  Children and Adolescents  -DCD Questionnaire (http://www.  dcdq.ca)  -MABC  (Henderson et al. 2007) | **MRI acquisition**: high-resolution (1.07 × 1.07 × 1.2 mm) T1 weighted  volumetric structural image  on a 3 T General Electric Signa scanner  (USA) using an eight-channel head coil | Motor coordination skill of aiming/catching significantly linked to latent variables for cerebral  cortex (t = 4.31, p < 0.0001) and the cerebellum (t = 2.31, p = 0.02), effect associated with premotor/motor cortical  regions and the superior cerebellar lobules.  DCD: atypical ↓ volumes of premotor/motor cortical  regions and the superior cerebellar lobules  DCD not significantly different from DCD + ADHD  Effect not moderated by severity of ADHD | Design of the study: not possible stating cause/effect, absence of follow-up  Latent variable is not directly observable but estimated from multiple evaluations.  Latent volume variables for wide regions, possibly hiding more specific regional variations |
| Bonthrone et al., 2024 | Cross-sectional  Case-control | | DCD: 36  1.DCD + Speech and language disorder 10;  2.DCD + dyslexia 10;  3.DCD + ASD or autistic traits 6; 4. DCD + ADHD 10;  TDC: 17 | | 8-10 yrs | - DSM-5  -MABC-II  - CCC-2  -questionnaires of executive unctioning and symptoms of inattention and hyperactivity | **MRI acquisition:**  T1-weighted and diffusion-weighted images | DCD:  - impaired attention, non-motor processing speed, and executive functioning  - cortical morphology in the posterior cingulate associated with both gross motor skills and inattentive symptoms  -gross motor skills associated with left corticospinal tract (CST) morphology | -Mixed sample with wide spectrum of co-occurring disorders  -ADHD medication influence on MRI  -Children who received improving motor intervention not excluded |
| Grohs et al., 2021 | Cross-sectional | | DCD 37  (12 children with ADHD)  TDC 48 | | 8-12 yrs | DSM-5  MABC-II  ADHD features: in-house parent questionnaire in supplementary materials | **MRI acquisition:**  T1-weighted and diffusion-weighted images  Brain volumes of the thalamus, basal ganglia, cerebellum and primary motor and sensory cortices were extracted using the FreeSurfer recon-all pipeline | ↓ volumes within both the left and right pallidum (Left: F = 4.43, p = 0.039; Right: F = 5.24, p = 0.025) | -Not possible stating cause/effect  - Small sample size  - Clinical heterogeneity within the sample  - reduced sensibility for subtle alterations  - Size information, difficult assessing the cerebral function |
| Fernandez et al, 2022 | Cross-sectional structural MRI study | | 252 children  ASD: 58  ADHD: 86  DCD: 22  TDC: 86 | | 7–12 yrs | DCD: MABC-2  ADHD: Conners3 ASD: ADOS-G / ADOS-2  - Motor performance assessed within cohorts | **T1-weighted structural MRI**; cerebellar voxel-based morphometry using ACAPULCO segmentation pipeline; voxel-wise GLM | ADHD group showed significantly reduced grey matter volume in the left Crus I compared to controls. No significant cerebellar grey matter volume differences were observed in children with DCD or ASD compared to controls. | -Small DCD sample size  - lack of harmonized behavioral data across cohorts  - cross-sectional design  - focus restricted to cerebellar macrostructure (no cortical or microstructural measures). |
| Butera et al, 2025 | Cross-sectional experimental study | | ASD: 18  DCD: 16  TDC: 20 | | 8–17 yrs | -MABC-2  -FAB-M  -ADOS-2 /ADI-R  -ADHD symptoms assessed via standardized parent-report questionnaires  -5-minute smart-tablet coloring task with kinematic data acquisition via touchscreen and inertial sensors | -Task-based fMRI during action execution and imitation  - Machine learning analysis of kinematic features from smart-tablet gameplay (touch and IMU sensors)  - Whole-brain voxel-wise and ROI analyses, focused on cerebellar Crus I and Crus II  - Correlations between kinematic features and cerebellar activation | -Standard motor assessments: not differentiate ASD and DCD  -Machine-learning analysis of kinematic features:  ASD vs DCD: 71% accuracy  ASD vs TDC: 76% accuracy  DCD vs TDC: 78% accuracy  -Task-based fMRI (action imitation): reduced activation of cerebellar Crus I and Crus II in children with DCD compared to ASD and TD  - Brain–behavior correlations: kinematic markers significantly correlated with cerebellar activity | -Small sample size;  - cross-sectional design; - wide age range;  - Machine learning findings require replication in larger cohorts  - MRI tasks performed separately from the smart-tablet task  - Limited generalizability to clinical settings  - Comorbidities and medication effects not fully disentangled |
| Kilroy et al., 2022 | Cross-sectional fMRI study | | DCD: 28 ASD: 33  TDC: 35 | | 8-17 yrs | -MABC-2  -ADOS-2/ ADI-R   -SRS-2  -NEPSY-II  - CONNERS 3 | MRI: task-based fMRI (3T scanner)  Tasks: action observation, action execution, imitation, mentalizing  Analyses: whole-brain voxel-wise analyses; ROI analyses focused on IFG pars opercularis (IFGop) and dmPFC; correlations with motor and social measures | - ASD: unique hypoactivation of right IFGop during action observation (embodied simulation deficit)  - ASD - DCD: IFGop hypoactivation during imitation (motor production)  -Across groups: IFGop activity correlated with motor ability (MABC-2) independent of social impairment  Mentalizing: dmPFC activity correlated with ToM ability, independent of motor skills | -Moderate sample size;  -cross-sectional design;  -wide age range;  - medication use present in clinical groups;  - fMRI task-based design limits ecological validity |
| Kilroy et al, 2022 | Cross-sectional comparative neuroimaging study | | DCD: 16  ASD: 22  TDC: 21 | | 8–17 yrs | MABC-2, DCDQ;  Florida Apraxia Battery–Modified ADOS-2, ADI-R. SRS-2,  AQC **Comorbidities**: ADHD (Conners 3), anxiety allowed; other neuro/psychiatric disorders excluded. | Diffusion-weighted MRI;  whole-brain correlational tractography using DSI Studio. ROI analysis on cingulum U-fibers; FDR-corrected permutation testing**.** | **-**ASD: white matter alterations in the fronto-parietal and parolfactory cingulum, forceps minor/anterior commissure, middle cerebellar peduncle, and right posterior IFOF/EC; associated with autism severity and alexithymia, but not with motor performance.  - DCD: alterations predominantly involving motor pathways (corticospinal and corticopontine tracts), associated with praxis abilities and motor scores.  Evidence of a dissociation between motor-related white matter abnormalities and those linked to core ASD symptoms. | - Small sample size per group;  -inclusion limited to high-functioning, right-handed participants  - Cross-sectional design  -Heavy reliance on parent/self-report measures for some domains. -Laterality effects may not generalize to left-handed individuals. |
| McLeod et al., 2014 | Observational case-control study using resting-state **functional MRI** | | DCD: 7  ADHD: 21  DCD + ADHD: 18  TD: 23 | | 7-12 yrs (mean age 9.4 yrs) | DSM-V  MABC  DCD-Q  DICA-IV  Conners’ Parent Rating Scale–Revised (CPRS-R) | Resting-state fMRI collected to examine intrinsic **functional connectivity of motor networks** (primary motor cortex, supplementary motor area, etc.);  Between-group comparisons of connectivity strength;  Correlation of connectivity metrics with motor performance measures. | **DCD only**: ↓ functional connectivity (FC) between the primary motor cortex (M1) and the caudate, putamen, globus pallidus, inferior frontal gyrus, posterior insular cortex, and prefrontal cortex.  **ADHD only**: ↓ FC between M1 and the striatum, putamen, inferior frontal gyri, left postcentral gyrus, frontal eye fields, and insular cortex.  **DCD + ADHD**: ↓ FC between M1 and somatosensory cortices, the left supramarginal gyrus, striatum, and amygdala, suggesting poor sensorimotor integration and impaired motor regulation.  **N.B:** compared to DCD or ADHD group, ↑ FC between M1 and the bilateral caudate, left premotor cortex, inferior frontal gyri, superior temporal gyri, precuneus cortices, and angular gyri. | Cross-sectional study: causal relationships cannot be determined.  Small sample size.  Age and brain development: FC differences may vary with maturation.  rs-fMRI does not capture structural or direct connectivity. |
| Yeh et al, 2012 | Observational  case-control | | 10 drug-naïve ADHD without DCD and 5 ADHD + DCD | | 15–18 yrs (mean ≈ 15.5 yrs) | -DSM-IV  -Movement Assessment Battery for Children-2 (≥95th percentile impairment) **-** Purdue Pegboard Test | 99mTc-ECD SPECT (resting-state rCBF) .  Voxel-wise analysis using SPM99 Threshold p < 0.001 (cluster-level correction) | Lower rCBF of bilateral frontal lobe, inferior parental lobe, and increased rCBF of right posterior cingulate gyrus, anterior lobe of cerebellum were found in ADHD comorbid DCD group compared to ADHD without DCD group. Decreased rCBF in the right occipital, inferior temporal lobe was found in ADHD comorbid DCD group after MPH while ADHD alone group revealed increased rCBF in bilateral occipital lobe. | - Very small sample  - No healthy control group - No ADHD subtype comparison - Acute MPH only (no long-term effects) - SPM conservative approach (possible false negatives) - No IQ assessment - Resting-state only |
| \| **Nemmi et al. al. 2023** \| \| --- \| | Cross-sectional  study | | TDC: 42 DD: 45 DCD: 20  DD+DCD: 29;  Total = 136 | | 8–12 years | **DCD:** M-ABC <5th percentile; DSM-5 criteria; ongoing motor therapy; IQ >70.  **DD:** Alouette test + ODEDYS-1 (≤1–1.5 SD below mean); DSM-5 criteria; speech therapy. | Structural MRI  Voxel-wise ANOVA (p<.001 uncorrected, cluster >50 voxels). | No single imaging index discriminated DD, DCD, and DD+DCD  Functional model (fALFF + connectivity) significantly discriminated groups (RF p = .04).  Complete model (structural + functional) showed best performance (RF p = .002; SVC p = .02).  **DCD:** discriminated mainly by cerebellar (Crus I/II, lobule VIII) and right MFG/IFG functional alterations (sensorimotor/fronto-parietal networks).  **DD:** discriminated by GMV in right superior temporal gyrus and right anterior insula.   **DD+DCD:** showed subtle but specific brain pattern, not purely additive of DD+DCD. | - Oversampling may increase overfitting risk;  - small DCD sample;  -voxel-wise threshold uncorrected;  -exclusion of ADHD limits generalizability;  - no DTI or advanced graph metrics; no direct brain  –behavior correlation analyses;  - cross-sectional design. |
| Kashiwagi et al.2009 | Case–control study | | DCD: 12  TD: 12 | | 9-12  years | M-ABC  **Soft Neurological Signs**  **Parent interview on daily motor activities**  **DSM-IV**  **WISC-III**  **Raven's Coloured Progressive Matrices** | Behavioural measures: tracking distance (pixels); velocity change (pixels/s/s)  Two-factor nested ANOVA; two-way factorial ANOVA  Task-based fMRI (visuomotor tracking paradigm)  Voxel-wise analysis using SPM5  Threshold z > 3.09 (P < 0.001 voxel-level); P < 0.05 cluster-level corrected  Random-effects group analysis  Correlation analysis (MR signal change vs task performance) | DCD :poorer task performance (greater distance and velocity change). Reduced activation in left posterior parietal cortex (superior and inferior parietal lobule, BA7, BA40) and left postcentral gyrus (BA2) in DCD vs controls during tracking.  No group differences in watching vs resting contrast. Negative correlation between MR signal change in left inferior parietal lobe and task performance (r = –0.413, P < 0.05).  Motor control deficit in DCD is present at cortical level (left PPC and postcentral gyrus), not basal ganglia or cerebellum. | -Very small sample size  - Inclusion of comorbidities within the DCD group (ADHD; developmental dyslexia) |
| Licari et al. 2015 | Case–control fMRI study | | DCD: 13  TDC: 13 | | 8–10 yrs | -MABC-2  -Zurich Neuromotor Assessment  -Vanderbilt ADHD Diagnostic Parent Rating Scale | 3T fMRI  Tasks: finger sequencing + hand clenching  Motion sensor glove per overflow Preprocessing: motion correction, smoothing 8mm  Random-effects GLM; FDR p<0.05; k>15 | **DCD:** greater contralateral motor overflow on both tasks; decreased activation in left superior frontal gyrus (BA9) and left inferior frontal gyrus (BA44) during finger sequencing;  increased activation in right postcentral gyrus (BA3) during finger sequencing;  no significant group differences during hand clenching;  no clear cortical activation deficits explaining abundant motor overflow.  **TDC:** lower contralateral motor overflow;  greater activation than DCD in left superior frontal gyrus (BA9) and left inferior frontal gyrus (BA44) during finger sequencing. | -Scan volume did not extend into subcortical areas.  -Motor overflow also present during hand-clenching contrast task, potentially limiting identification of overflow-specific regions.  -Selection based on one DSM-IV criterion |
