# Supplementary material for: Advancing understanding of developmental coordination disorder in children: data from the literature
Source: Front Hum Neurosci. 2026 Jun 3;20:1779776. doi: 10.3389/fnhum.2026.1779776 (PMC13272446; doi:10.3389/fnhum.2026.1779776)
Supplement: Supplementary file 2 [file Table_2.docx]

| Study, Author, Year | | Design | | Population | | Age | | Assessment for  DCD/Motor Performance | | Analyzing methods | | Main Findings  (DCD vs TCD) | | Limitations | |
| --- | --- | --- | --- | --- | --- | --- | --- | --- | --- | --- | --- | --- | --- | --- | --- |
| Lum et al., 2025 | | Cross-sectional case-control study | | DCD: 31  TDC: 52 | | 9.5 yrs | | DSM 5  Test of Motor Proficiency 2nd edition (BOT- 2 SF); “Matrix Reasoning” and “Vocabulary” subtests from the Wechsler Abbreviated Scale of Intelligence- 2nd Edition (WASI- II); | | Resting-state EEG (eyes open/closed, 23 electrodes, 10/20 montage) | | DCD exhibited lower alpha power and higher delta power at rest compared to typically TCD. No significant differences in beta power were found between the groups**.** | | - Unclear mechanism of beta abnormality  - Small DCD sample  - EEG spatial resolution  - Cross-sectional design | |
| Blais et al. 2018 | | Experimental  EEG study | | DCD: 10  TCD: 10 | | 12-16 yrs | | Bimanual coordination task (in-phase, anti-phase, novel) | | EEG coherence analysis (fronto-central); Behavioral measures (accuracy, stability, mirror and parallel movements) | | **DCD+TCD**: improved accuracy after practice, associated with ↑ right intrahemispheric coherence  **DCD**: less stable execution, more mirror movements, significantly ↓ interhemispheric coherence | | Small sample  Restricted age  Limited generalizability | |
| De Castelnau et al, 2008 | | Cross-sectional experimental EEG study | | DCD: 24  TDC: 24 | | 8-13 yrs | | Clinical diagnosis of DCD, supported by standardized motor assessment (M-ABC2).  Motor task: bimanual motor synchronization task performed in time. | | EEG; coherence analysis between cortical regions during a motor synchronization task. | | DCD: altered EEG coherence patterns during motor synchronization, particularly ↓ inter-and intra-hemispheric coherence in motor-related regions. | | - Small simple size  - Cross-sectional design  - Limited spatial resolution of EEG  - Lack of detailed assessment of comorbid neurodevelopmental conditions. | |
| Pangelinan et al, 2013 | | Cross-sectional observational case-control study using EEG | | DCD: 14  TDC: 20 | | 6-12 yrs | | Clinical diagnosis of DCD, supported by standardized motor assessment (M-ABC2).  Motor performance was evaluated through task-specific behavioral measures during experimental motor tasks. | | EEG during the execution of goal-directed motor tasks.  Movement-related cortical potentials (MRCPs) were analyzed to examine the timing and amplitude of cortical activation preceding and during movement execution. | | DCD: atypical movement-related cortical activation patterns 🡪 altered amplitude and timing of MRCPs.  Poorer motor performance. | | - Small sample size  - Cross-sectional design  - Limited spatial resolution of EEG  - Results were based on specific motor tasks | |
| Boćkowski, 2005 | | Cross-sectional observational study using EEG | | DCD: 2 | | 5 and 16 yrs | | Clinical diagnosis of DCD, supported by standardized motor assessment (M-ABC2).  Motor performance was evaluated through task-specific behavioral measures during experimental motor tasks. | | Cortical evoked potentials (EPs) were recorded using EEG in response to somatosensory or motor stimulation | | DCD: altered cortical evocated potentials, including differences in amplitude and latency compared to TD children | | - Sample size not clearly specified  - Cross-sectional design  - Limited methodological detail on task and EEG parameters | |
| Tsai et al, 2009 | | Case-control study  EEG study | | DCD: 28  TDC: 26 | | 9-10 yrs | | - M-ABC  - BOT-2-SF  - WISC—R  -DSM-IV | | Reaction times  (RTs) to each target  EEG analysis for baseline-to-peak amplitudes and  cue-to-peak or target-to-peak latencies | | - **DCD** responded significantly more slowly than typically developing children and exhibited a deficit of inhibitory  response capacity in the endogenous mode of orienting attention  **- DCD**: slower target identification, less ability in interhemispheric and cognitive-tomotor transfer speed, and a less mature anticipatory and execution  processes. | | ND | |
| Tsai et al, 2012 | | Quasi-experimental longitudinal intervention study | | DCD: 40 (20 training, 20 non-training)  TDC: 20 | | 11-12 yrs | | MABC 2  DSM-IV | | DCD training group: exercise intervention (vs TDC and DCD non-training).  Motor Assessment (MABC).  Cardiorespiratory fitness (PACER)  Cognitive Task (VSWM)  EEG | | Baseline  DCD (training and no training) had lower cardiorespiratory fitness, poorer motor performance (MABC-2), lower accuracy and slower RTs in delayed VSWM conditions, reduced P3 and pSW amplitudes during retrieval compared to TD children  Post Training  DCD training group improved in Cardiorespiratory fitness, motor skills (MABC-2), VSWM accuracy (3s and 6s delays), P3 amplitude during encoding and retrieval (similar to TDC) | | Lack of a placebo or active control intervention.  Limited assessment of broader functional outcomes.  No follow-up or wash-out period.  Constraints related to neurological characteristics of DCD. | |
| Wang et al, 2017 | | Cross-sectional case controlled study | | DCD: 29  TDC: 29 | | 10-11 yrs | | MABC2  DSM 5 | | Motor Assessment (MBAC 2)  Cognitive task (VSWM)  EEG | | Delayed condition DCD group showed lower accuracy and slower RTs than TD  Non delayed no difference  EEG reduced theta increases in DCD in encoding and retrieval  EEG reduced alpha suppression in late maintenance phase | | IQ Characteristics  Small sample size  Limited sensitivity of EEG–behavior correlations | |
| Wang et al, 2015 | | Cross-sectional case controlled study | | DCD: 23  TDC: 23 | | 9-10 yrs | | MABC2  DSM-IV | | Motor Assessment  (M-ABC2)  Cognitive/attentional task  EEG | | DCD group shows deficits in attentional orienting, reflected in slower behavioral responses and in reduced frontal midline theta modulation.  Theta oscillations index top-down attentional control and sensorimotor integration.  Frontal midline theta is proposed as a neurophysiological marker of attentional deficits in DCD. | | ND | |
| Song et al, 2025 | | Cross-sectional case-control characterization study | | DCD: 15  TDC: 15 | | 6-8 yrs | | M-ABC2 | | Visual-motor integration task  EEG | | Motor coordination difficulties in DCD boys are associated with: reduced functional brain connectivity and abnormal hemispheric lateralization.  Connectivity and lateralization abnormalities, rather than resting EEG power or signal complexity, may underlie DCD motor deficits. | | - Small sample size  - Cross-sectional design  - Restricted sample characteristics (only boys, only right-handed participants.  - Diagnostic limitations (only MABC-2 | |
| Lust et al, 2019 | | Cross-sectional case-control study | | DCD: 14  TDC: 14 | | 9-13 yrs | | DSM-V  MABC-2  DCD-Q | | - Motor assessment: MABC-2  - Action observation/imitation task: sequential pointing task  - EEG | | DCD: neurophysiological evidence of mirror neuron system dysfunction (significantly more imitation errors than TDC)  Impairments reflect a reduced activation (mu suppression) and a reduced integration of action goals and means (mu coherence). These deficits likely contribute to poor observational motor learning and difficulties acquiring new motor skills | | - Interpretation of mu rhythm.  - Lack of precise source localization of neural generators.  - Small sample size.  - Cross-sectional design.  - Task specifity | |
| Chang et al, 2021 | | Cross-sectional case-control study | | Behavioral experiment  DCD: 20  TDC: 27  EEG experiment  DCD: 27  TDC: 27  Samples partially overlapped: 40 children completed both experiments | | 6-7 yrs | | MABC-2  Parent interview  KBIT-2  CDC ADHD checklist | | - Behavioral psychophysivcal tasks: three 2-alternative forced choice tasks using adaptive staircases.  - EEG | | - Auditory perceptual timing deficits are present in children with motor difficulties  -Timing deficits are not secondary to motor execution, as tasks were non-motor  - Suggests auditory–motor timing dysfunction may be core to DCD pathophysiology | | -Small sample size.  - Cross sectional design  - No formal clinical diagnosis  - Behavioral and EEG data non collected simultaneously.  - Potential contribution of latent factors | |
| Warlop, 2025 | | Observational case-control | | DCD: 21  TCD: 15 | | 12-15 yrs | | DSM-IV  MABC 2 | | EHI for hand dominance  Pittsburg Sleep Quality Index  EEG for visuomotor adaptation experiment  Accelerometer for sleep quality/quantity  Questionnaire: DCD-Q, VvGK6-16, Dutch version of SRS-2, Dutch version of BRIEF  400 trials total  PSQI questionnaire for sleep evaluation | | TD participants almost never scored in the higher range of the component scores (scores 2 and 3), while participants with DCD did (PSQI)  7 participants with DCD had a global PSQI score over 5, whereas this was only the case in 3 of the TD participants  DCD having poorer self-reported sleep efficiency than the TD group  Larger directional error prior to adaptation in the DCD group compared to the TD group  On day 1, slightly, yet not significantly, larger directional error during early adaptation in the DCD group  When comparing the final level of adaptation between the groups, the ANOVA indicated a significantly larger directional error in the DCD group Vs TD  After removal of the rotation, during the post-exposure phase, both groups showed significantly larger directional error  Some adaptation from day 1 to day 2  No significant difference in EEG | | ND | |
| Warlop et al., 2024 | | Case-control | | DCD: 33  TCD: 33 | | 13.0 ± 2.0 yrs | | -Already received  diagnosis of DCD (DSM-V)  -MABC-II | | EEG frequency tagging and apparent biological motion (Cracco et al., 2023) | | DCD: no ↓ sensitivity to apparent biological motion, compared with TCD children.  DCD: ↓ brain response to repetitive visual stimuli, indicating altered predictive processing in the perceptual domain | | Not definitively  describe the altered mechanism of the perception of biological motion | |
| Fong et al., 2016 | | Cross-sectional observational EEG study | | DCD: 86  TCD: 99 | | 12-16 yrs | | MABC | | EEG (NeuroSky)  Attention index (0–100) derived from weighted EEG bands, recorded during tasks | | DCD: ↓ MABC scores,  ↓ attention index vs TCD; attention index correlated with motor → motor deficits linked to attentional processes. | | 1-channel EEG  cross-sectional design  lack of detailed age/confounder data.  Presence of ADHD in some participants | |
| Gomez et al., 2017 | | Case-control | | 20 children with and 20 children without DCD | | - 1. years | | -Already received  diagnosis of DCD  -DSM-IV-TR | | Eye-tracking (Tobii TX-300 ey) | | Latency of response and lower grade of precision | | -difficult understanding mechanisms underlying mathematical learning difficulties in children with **DCD** and in  children **without DCD**  - only focus on ability from **DCD** group to  comprehend the concept of linearity which underlie mathematical analysis | |
| Sumner et al., 2018 | | Case-control | | 77 children, 3 groups:  - DCD children (aged 7–10),  -chronologically age (CA) matched peers,  -motor-match (MM) group (aged 4–7). | | 7-10; 4-7 years | | MABC-2 (second version) | | -Eye-tracking technology, visual fixation, smooth pursuit, and pro- and anti-saccade performance.  Eyelink 1000 eye tracker (SR-research) | | **DCD children** showed problems with regards to saccadic inhibition and maintaining attention on a visual target. | | -small sample for each analyzed group  Mixed findings reported of visual deficits in DCD. | |
| Robert et al, 2014 | | Cross-sectional case-control study | | DCD: 27  TDC: 64 | | 7-12 yrs | | DSM-IV TR | | Electro-oculography: horizontal smooth pursuit gain and vertical smooth pursuit gain. | | DCD: vertical smooth pursuit selectively impaired. It supports a delayed maturation of the oculomotor pursuit system. Results are consistent with cerebellar dysfunction and cerebello–cerebral network involvement in DCD | | -Cross-sectional design  - Heterogeneity of intervention | |
| Ferguson et al, 2015 | | Cross-sectional case-control study | | DCD: 40  TDC: 40 | | 6-10 yrs | | DSM-IV | | Visual-manual tracking task | | DCD: marked difficulties in visuo-manual tracking. Deficits are evident when visual feedback is reduced and predictive control I required. Results support models implicating of cerebellar dysfunction and parietal cortex involvement.  Predictive control deficits may underlie everyday motor difficulties in DCD | | - Comorbidity non formally assessed  - Cross-sectional design  - Mechanistic ambiguity | |
| Licari et al., 2018 | | Case-control | | 24 boys: 11 with DCD and 13 controls | | -DCD 9.43 years±0.73  -controls (9.16 years±0.68) | | -American Psychiatric Association, 2013 (Criterion A, B, C, D)  -MABC-2 (second version) | | Eye-tracking: Spectacle Mounted Unit (SMU), the Rear Mounted Unit (RMU) and a  laptop computer. | | -Prior to ball release: **DCD group** more fixations (p=0.043) of  inferior duration (p=0.045).  -Phase of flight: **DCD group** longer to start smooth pursuit  (p=0.003). After the induction of movement, adequate ability of maintaining smooth  Pursuit in both categories. The opening delay does not affect the initiation of the movement initiation time (p=0.173). Nevertheless,  Time of motion relevantly slower in the **DCD group** (p=0.031). | | -small sample  -only males’ sample | |
| Miles et al, 2015 | | Randomized controlled intervention study | | DCD 30  - Quiet eye training (QET) 15  - Traditional training 15  NO TDC | | 8-10 yrs | | -Clinical diagnosis (occupational therapist)  - MABC-2  -Parent and teacher report  - ADHD Rating Scala-IV | | Motor assessment  Visuomotor task  Eye tracking  Kinematic measures | | Quiet Eye Training is learnable by children with DCD, durable over time and more effective than traditional instruction for improving visuomotor coordination. Improvements likely reflect a better attentional control and an improved prediction of ball flight. QET may be a valuable adjunct to therapy and education for children with DCD | | - Short intervention duration  - Binary success measure sensitivity  - Individual variability  - Mechanistic ambiguity  - Task specificity | |
| Wood et al., 2017 | | Randomized controlled trial | | 1.Quiet eye training, QET group (8 male 3 female, mean age of 8.6 years  2.Technical training, TT group (7 male 3 female, mean age of 8.6 years | | 7-11 years | | - MABC-2 (second version) | | -Eye-tracking: Quiet Eye Solutions software (www.  quieteyesolutions.com)  -Gait Analysis: Sagittal motor video footage | | -**QET group** improve gaze control and catching coordination  -longer QE aiming duration (QE1) is associated to a previous start of tracking the target prior to catching (QE2), also connected to catching success. | | -use of unvalidated parental  feedback questionnaire and a relatively small sample size. | |
| Parr et al, 2020 | | Cross-sectional case-control study | | DCD: 21  TDC: 18 | | 8-15 yrs | | DSM-V | | - Adaptive locomotion task: step accurately into a raised rectangular target box, then step over no obstacle, one obstacle and two obstacles.  - Kinematic measures: 3D motion capture  - Eye tracking  - Anxiety measure: child-friendly fear thermometer | | DCD: reduced foot placement accuracy and precision. Deficits occur independently of gaze behavior and state anxiety. Findings suggest general neuromuscular control deficits and reduced ability to rapidly integrate multisensory information. Foot placement deficits may partially explain increased fall risk in DCD | | - Small and heterogeneous sample  - Anxiety measurement limitations  - Low task-related anxiety  -Covert attention not measured | |
| Bellocchi et al., 2022 | | Case-control and comparison between groups with different clinical features | | 138 children (57 females and 81 males) | | 7 years and 8 months – 12 years and 6 months | | - French version 242 of the M-ABC (Soppelsa & Albaret, 2004) | | Eye-tracking | | -**Unique DCD**: varies from TD only for the errors’ z-score (p<.039).  -**Children with DD**: lower z-scores than TD children for the vertical time, horizontal time, and as regards the errors.  -**DCD+DD**: more inferior z-scores than TD as regards the vertical time, horizontal time, errors and for the ratio. | | -different size sample within the different analyzed group  - DEM test does not highlight specific oculomotor alteration within the DCD group | |
| Sumner E. et al, 2018 | | Cross-sectional observational study using **eye-tracking** | | DCD: 28  ASD: 28  TD: 26  Total: 82 children | | 7-10 years | | WASI-II (IQ ≥ 80)  MABC-2: DCD group scored ≤ 16th pc; TD group scored ≥ 25th pc.  SCQ (cut-off 15)  ADOS-2 | | Eye movements were recorded with a **Tobii X2-60 eye tracker** while children viewed **30 static social images**. Analyses focused on **fixation time**, **time to first fixation** on faces, and **gaze-following** toward objects of interest. **AOIs** included faces, eyes, and socially relevant objects. | | **DCD**: intermediate attention to faces and eyes (↓ than TD, ↑ than ASD).  Time to first fixation on faces was similar to TD.  ↓ Gaze-following compared to TD: lower spontaneous sensitivity to social cues.  **ASD:** ↓ time looking at faces and eyes than TD.  ↓ gaze-following compared to TD.  **TD:** Typical attention to faces and eyes, faster first fixation, and ↑ gaze-following.  ↑ SCQ scores correlated with less face fixation  Motor abilities (MABC-2) showed no significant relationship with social attention | | Cross-sectional study: causal relationships cannot be determined.  Static images: no caption of complexity and dynamics of real social interactions or videos.  Small sample size.  Group differences: despite inclusion criterion of IQ ≥ 80, TD children tended to have slightly higher IQ than clinical groups; some DCD children had elevated SCQ scores, suggesting possible overlap with ASD symptoms.  Eye-tracking: does not reveal how children interpret social cues (e.g., understanding emotions, intentions, or social rules). | |
| Woodruff et al, 2002 | | Observational study | | DCD. 7  TCD: San Diego Database | | 6-7 yrs  San Diego Database: 3-7 yrs | | MABC Checklist and Test  (Henderson & Sugden, 1992) | | Index of Walking Performance: one-dimensional evaluation of  normal gait pattern using time/distance gait variables | | - DCD children: prevalence of altered walking patterns.  - Means of the time/distance gait variables did not differ between children with DCD and San Diego children.  - DCD children had much larger differences than other children, suggesting no systematic pattern in individual pattern.  differences. | | Small sample size | |
| Chia, 2013 | | Cross sectional study | | DCD: 30  TCD: 142 | | 6-12 yrs | | Japanese  version of the Strengths and Difficulties Questionnaire (SDQ-J) | | Gaith analysis  Step length and step time  GDI symmetry ratio, step length, and step time symmetry ratios | | - DCD trait had lower  total and three subscale DCDQ-J scores (p < 0.0001) and higher SDQ-J  scores than those without DCD trait (p < 0.001)  - DCD trait had a lower GDI score (p < 0.0001) and a higher GDI symmetry ratio (p = 0.004) than those without DCD trait  - In the logistic  regression analysis, there was a significant relationship between DCD  trait and GDI symmetry ratio  - The step length and step time  symmetry ratios were not significantly associated with DCD trait  **-** fine motor, handwriting, and general coordination  scores were not significantly correlated with the GDI symmetry ratio  - a reduced and  bilateral asymmetric gait quality is one of the main gait characteristics in  those with DCD trait. | | ND | |
| Deconinck et al, 2006 | | Cross-sectional case-control study | | DCD: 10  TDC: 10 | | 6-8 yrs | | DSM IV | | Gait Analysis | | Gait differences reflect adaptive strategies, not random abnormality.  Children with DCD appear to prioritize stability, safety and reduced destabilizing forces.  Findings consistent with neuromuscular immaturity, postural control deficits and sensory integration difficulties | | - Small sample size  - Treadmill walking paradigm  - Lack of kinetic and EMG data  - Sensory contributions inferred, not measured | |
| Stewart C. Morrison, 2013 | | Randomized clinical trial | | 14 children | | 6-11 years | | Individual with already assessed DCD diagnosis, (Physical and Developmental  Assessment rehabilitation programme within the Children’s Therapy Service at Medway Community Healthcare  [- Foot Posture Index  - Lower Limb Assessment Score] | | Gait Analysis: the GAITRite walkway®, utilized for the evaluation of spatio-temporal gait variables | | No-significant results related to spatio-temporal gait variables (p > .012) | | -Small sample size  -No-relevant outcomes | |
| Ito, 2021 | | Cross-sectional case-control study | | DCD: 14  TDC: 14 | | 7-10 yrs | | DSM IV  MABC 2 | | Average running speed  Running Kinematics | | DCD displayed reduced individual peak knee extension immediately prior to initial foot contact.  No interaction in knee, ankle or hip DCD group displayed increased variability in the sagittal plane kinematics of the hip and ankle during toe off compared with the TD group (variability of hip extension angle during toe off, of plantar flexion angle during toe off, foot progression angle throughout the stance and swing phase compared  with the TD children, particularly after toe off)  DCD reduced peak knee extensor moment during the stance phase | | Male-only sample | |
| Goetschalckx, 2024 | | Observational cross-sectional case-controlled study | | DCD: 21  TDC: 23 | | 8-12 yrs | | MABC2  DSM 5  DCD Questionnaire | | Motor performance: gross and fine motor skills, and postural control (MABC-2)  Continuous three-minutes walking and running  DCDQ  Kids BESTest  Interlimb coordination | | Children with DCD  run with a significantly higher interlimb coordination (PCI) than their typically developing peers (less coordinated running pattern).  No difference while walking.  Only within the DCD group, the log(φCV) is significantly higher during running compared to walking.  Children with DCD run with a significantly lower absolute gait velocity and shorter absolute step length and flight time, and longer contact time.  DCD show a significantly higher variability in cadence, step length and gait velocity than TDC both during walking and running. | | ND | |
| Goetschalckx 2024 | | Observational cross-sectional case-controlled study | | DCD: 21  TDC: 22 | | 8-12 yrs | | DSM 5  MABC 2  DCDQ | | -Motor performance according to MABC 2.  -Rhythm and melody perception according to MBEMA-s.  -Executive functioning  Experimental paradigm  (auditory-motor synchronization, interlimb coordination, spatiotemporal gait variability) | | DCD: impaired auditory-motor synchronization consistency, reduced interlimb coordination and elevated gait variability. Deficits are amplified under slowed tempo constraints. | | ND | |
| Speedtsberg, 2018 | | Cross-sectional matched case-control study | | DCD: 8  TDC: 10 | | 7-11 yrs | | DSM IV  MABC 2 | | 4-minutes walking test: trunk acceleration, upper body accelerations in vertical (VT), medio-lateral (ML) and anterior-posterior (AP) directions | | No significant difference in  preferred walking speed between the children with DCD and TD.  The normalized RMS acceleration (RMSR) showed no difference between children with DCD and TD children in VT, ML or AP neither did the raw acceleration RMS.  Reduced local dynamic  stability in children with DCD than that of TD children in the AP direction.  When excluding the two  children with DCD that held onto the handlebars during treadmill walking, the difference in ML λs between children with DCD and TD  children reached statistical significance. | | Small sample size  Difficult to recruit  Narrow exclusion criteria | |
| Speedtsberg 2017 | | Cross-sectional case-control study | | DCD: 9  TDC: 10 | | 8-10 yrs | | DSM IV  MBAC 2 | | Static bipedal standing balance  Six sensory conditions were created by alternating firm or compliant support surface and visual input with the purpose of manipulating visual and proprioceptive feed-back and test the effect on the underlying mechanisms of postural control.  Unreliable vision was created using a half dome placed over child’s head  offering no reliable spatial information 3 trials in each of 6 conditions | | Impaired balance in DCD cannot be explained by central deficits alone.  Peripheral neuromuscular control plays a significant role.  Balance interventions for DCD should include tasks stressing feedback control and an altered proprioceptive conditions. | | - Small sample size | |
| Fong et al., 2016 | | Cross-sectional | | DCD: 30  TCD: 20 | | 6.10 yrs | | MABC | | Computorized dynamic posturoghraphy | | DCD children showed short limit of stability maximum excursion in the backward direction corresponding to higher number of falls in daily life. | | 1not blinded group, recal bias in reporting falls | |
| Beani et al, 2022 | | Cross-sectional comparative study | | DCD: 20 children  TDC: 30  Cerebral palsy: 27 | | 4-16 y | | -DSM 5 criteria  -MABC  -Gross Motor Function Classification System | | Virtual Reality Rehabilitation System (VRRS) | | -Cerebral Palsy group obtained the worst values.  -DCD group obtained intermediate values.  -Poorer motor skills are associated with greater postural instability. | | - No dynamic tasks.  - No sensory conditions  - Heterogeneous group | |
| Gentle et al, 2016 | | Cross-sectional matched case-control study | | DCD: 35  TDC: 35 | | 8-32 y | | -DSM 5 criteria  -MABC  - Bruininks Oseretsky Test of Motor Proficiency, Second Edition, Brief Form  - Strengths and difficulties questionnaire | | VICON 3D motion analysis | | All participants adopted a safer walking strategy on irregular terrain, but DCD group showed even greater adaptations to the irregular terrain, suggesting a reduced dynamic stability and a difficulty integrating multisensory information | | - Head angle as proxy for gaze  - Cross-sectional design  -Ecological validity still limited  -Sensory integration inferred, not directly measured  -Missing data due to marker occlusion | |
| Hsu et al, 2018 | | Cross-sectional case-control study | | DCD: 20  TDC: 20 | | 5-12 y | | -Developmental Coordination Disorder Questionnaire;  -MABC 2  -Motor Planning Maze Assessment  -Dynamic Gait Index  -Functional Gait Assessment | | Motor Planning test  Gait coordination test | | DCD: difficulties in motor planning skills and gait coordination | | - Small sample size  - Lack of assessor blinding  - Assessment tools are not standardized or age-correct  - Age effects not fully tested | |
| Wilmut et al., 2017 | | Case-control | | DCD: 62  TDC: 62 | | Years (44 individuals < 18 years | | -DSM-5 criteria for DCD and recent UK guidelines  - individuals < 18 years: MABC-2  - individuals > 18 years: combination of the MABC-2 and the Bruininks-Oseretsky Test of  Motor Proficiency, Second Edition, Brief form | | Measurement of step time and step length for both lower limbs ratios of symmetry | | **DCD**: increased ratios of symmetry, with around 30% of DCD group characterised by values outer the symmetry range.  Association between grade of asymmetry and variability of the movement. | | Only two outcome measures, step time and step width | |
| Beernaert et al, 2019 | | Cross-sectional case-control using qualitative trajectory calculus | | DCD: 9  TDC: 9 | | 6-8 | | - DSM IV criteria  - M-ABC2 | | Qualitative trajectory calculus applied to gait analysis | | Qualitative trajectory calculus can sistinguish DCD gait patterns with relatively high accuracy | | - Small sample size  - Use of treadmill walking  - Limited number of steps  - The experimental set-up is still expensive and complex for clinical use | |
